# Germline nuclear-predominant Pten murine model exhibits impaired social and perseverative behavior, microglial activation, and increased oxytocinergic activity

**DOI:** 10.1186/s13229-021-00448-4

**Published:** 2021-06-04

**Authors:** Nick Sarn, Stetson Thacker, Hyunpil Lee, Charis Eng

**Affiliations:** 1 Genomic Medicine Institute, Lerner Research Institute, Cleveland Clinic, 9500 Euclid Avenue, Cleveland, OH 44195 USA; 2 Department of Genetics and Genome Sciences, Case Western Reserve University School of Medicine, Cleveland, OH 44106 USA; 3 Cleveland Clinic Lerner College of Medicine, Case Western Reserve University, Cleveland, OH 44195 USA; 4 Germline High Risk Focus Group, Case Comprehensive Cancer Center, Case Western Reserve University School of Medicine, Cleveland, OH 44106 USA

**Keywords:** *PTEN* mutation, Autism spectrum disorder, Mouse model, Social impairment, Microglia, Complement, Neuroinflammation, Neurodegeneration, Oxytocin

## Abstract

**Background:**

Autism spectrum disorder (ASD) has a strong genetic etiology. Germline mutation in the tumor suppressor gene *PTEN* is one of the best described monogenic risk cases for ASD. Animal modeling of cell-specific *Pten* loss or mutation has provided insight into how disruptions to the function of PTEN affect neurodevelopment, neurobiology, and social behavior. As such, there is a growing need to understand more about how various aspects of PTEN activity and cell-compartment-specific functions, contribute to certain neurological or behavior phenotypes.

**Methods:**

To understand more about the relationship between Pten localization and downstream effects on neurophenotypes, we generated the nuclear-predominant *Pten*^*Y68H/*+^ mouse, which is identical to the genotype of some PTEN-ASD individuals. We subjected the *Pten*^*Y68H/*+^ mouse to morphological and behavioral phenotyping, including the three-chamber sociability, open field, rotarod, and marble burying tests. We subsequently performed in vivo and in vitro cellular phenotyping and concluded the work with a transcriptomic survey of the *Pten*^*Y68H/*+^ cortex, which profiled gene expression.

**Results:**

We observe a significant increase in P-Akt downstream of canonical Pten signaling, macrocephaly, decreased sociability, decreased preference for novel social stimuli, increased repetitive behavior, and increased thigmotaxis in *Pten*^*Y68H/*+^ six-week-old (P40) mice. In addition, we found significant microglial activation with increased expression of complement and neuroinflammatory proteins in vivo and in vitro accompanied by enhanced phagocytosis. These observations were subsequently validated with RNA-seq and qRT-PCR, which revealed overexpression of many genes involved in neuroinflammation and neuronal function, including oxytocin. Oxytocin transcript was fivefold overexpressed (*P* = 0.0018), and oxytocin protein was strongly overexpressed in the *Pten*^*Y68H/*+^ hypothalamus.

**Conclusions:**

The nuclear-predominant *Pten*^*Y68H/*+^ model has clarified that Pten dysfunction links to microglial pathology and this associates with increased Akt signaling. We also demonstrate that Pten dysfunction associates with changes in the oxytocin system, an important connection between a prominent ASD risk gene and a potent neuroendocrine regulator of social behavior. These cellular and molecular pathologies may related to the observed changes in social behavior. Ultimately, the findings from this work may reveal important biomarkers and/or novel therapeutic modalities that could be explored in individuals with germline mutations in *PTEN* with ASD.

**Supplementary Information:**

The online version contains supplementary material available at 10.1186/s13229-021-00448-4.

## Background

It is well established that germline mutations in *PTEN* predispose individuals to autism spectrum disorder (ASD) and rank among the most common monogenic etiologies [1–13]. We and others have shown that 7–20% of individuals with ASD and concurrent macrocephaly harbor germline mutations in *PTEN*, which when extrapolated across all cases of ASD may account for 0.5–5% of those cases [5, 7, 8, 13–15]. In addition to ASD, germline *PTEN* mutations cause subsets of Cowden syndrome (CS, OMIM #158350), Bannayan-Riley Ruvalcaba Syndrome (BRRS), Proteus syndrome (OMIM #176920), and Proteus-like syndrome [16]. Irrespective of clinical syndrome and pathophysiology, anyone diagnosed with a germline *PTEN* mutation carries the molecular diagnosis of PTEN Hamartoma Tumor Syndrome (PHTS, OMIM #601728) [16, 17].

PTEN has been well characterized as a tumor suppressor gene that removes the 3’ phosphate group from phosphatidylinositol(3,4,5)-triphosphate (PIP3), thereby inhibiting the PI3K/AKT/mTOR signaling pathway, a major growth, survival, and migration pathway [16, 18, 19]. Beyond this canonical PTEN function, there is a growing body of research exploring the protein phosphatase and non-catalytic activities of PTEN [20, 21]. The subcellular localization of PTEN and its importance to neurological phenotypes is of special interest since very little is known about PTEN function with respect to regulating these processes. However, recent work suggests changes in Pten subcellular localization (i.e., cytoplasmic predominance) may be important to neuronal and glial functions and may be associated more often with ASD phenotypes [22–24]. Moreover, it has been observed that missense mutation versus other types of mutation is enriched among individuals with ASD [25–28]. However, there are still outstanding questions about the exact impact of PTEN localization on neurological structure and function. There is a growing need to study and characterize *PTEN* mutations that affect PTEN localization and develop an understanding of how PTEN localization affects organismal phenotypes.

In order to interrogate the effects of PTEN mislocalization, we developed two complementary mouse models of germline *Pten* mutation that either simulates or copies PHTS genotypes. One model exhibits cytoplasmic-predominant localization of Pten, the *Pten*^*m3m4*^ model, while the other exhibits nuclear-enriched localization of Pten, the *Pten*^*Y68H*^ model. In the cytoplasmic-predominant model, we found no significant morphological or behavioral changes in *Pten*^*m3m4/*+^ mice, but in the homozygous *Pten*^*m3m4/m3m4*^ mice we observed dramatic macrocephaly and a sex-dependent increase in sociability with severe deficits in motor coordination [22]. Additionally, we performed extensive cellular phenotyping on the *Pten*^*m3m4*^ model, finding hypertrophy of neuronal somas, astrogliosis, dysmyelination, stunted maturation of neural stem cells (NSCs), precocious differentiation of oligodendrocyte progenitor cells (OPCs), and microgliosis, specifically cell-autonomous microglial activation and increased phagocytic response [22, 25, 29–31]. Our molecular characterization of the *Pten*^*m3m4*^ model included an RNA sequencing experiment that found that the neural transcriptome included many genes relevant to human idiopathic ASD, many of which were microglia-specific and associated with complement and neuroinflammatory pathways [23]. In contrast to the cytoplasmic-predominant model, the nuclear-predominant *Pten* mutant has never been characterized or subject to study. The Y68H mutation was initially observed in individuals with PTEN-ASD and favors nuclear localization of Pten [32, 33]. We suspect the mutation’s effect on Pten subcellular localization likely results in neurodevelopmental deficits, which may contribute the pathophysiology similar to that of PTEN-ASD. Therefore, we hypothesize that the nuclear-predominant *Pten*^*Y68H*^ mutation contributes to cellular deficiencies in the function of neurons and glia, associating with behavioral changes reminiscent of ASD. Here, we provide a broad characterization of *Pten*^*Y68H*^ mouse evaluating behavioral, cellular, and molecular phenotypes.

## Methods

### Animals

**Fig. 1:**
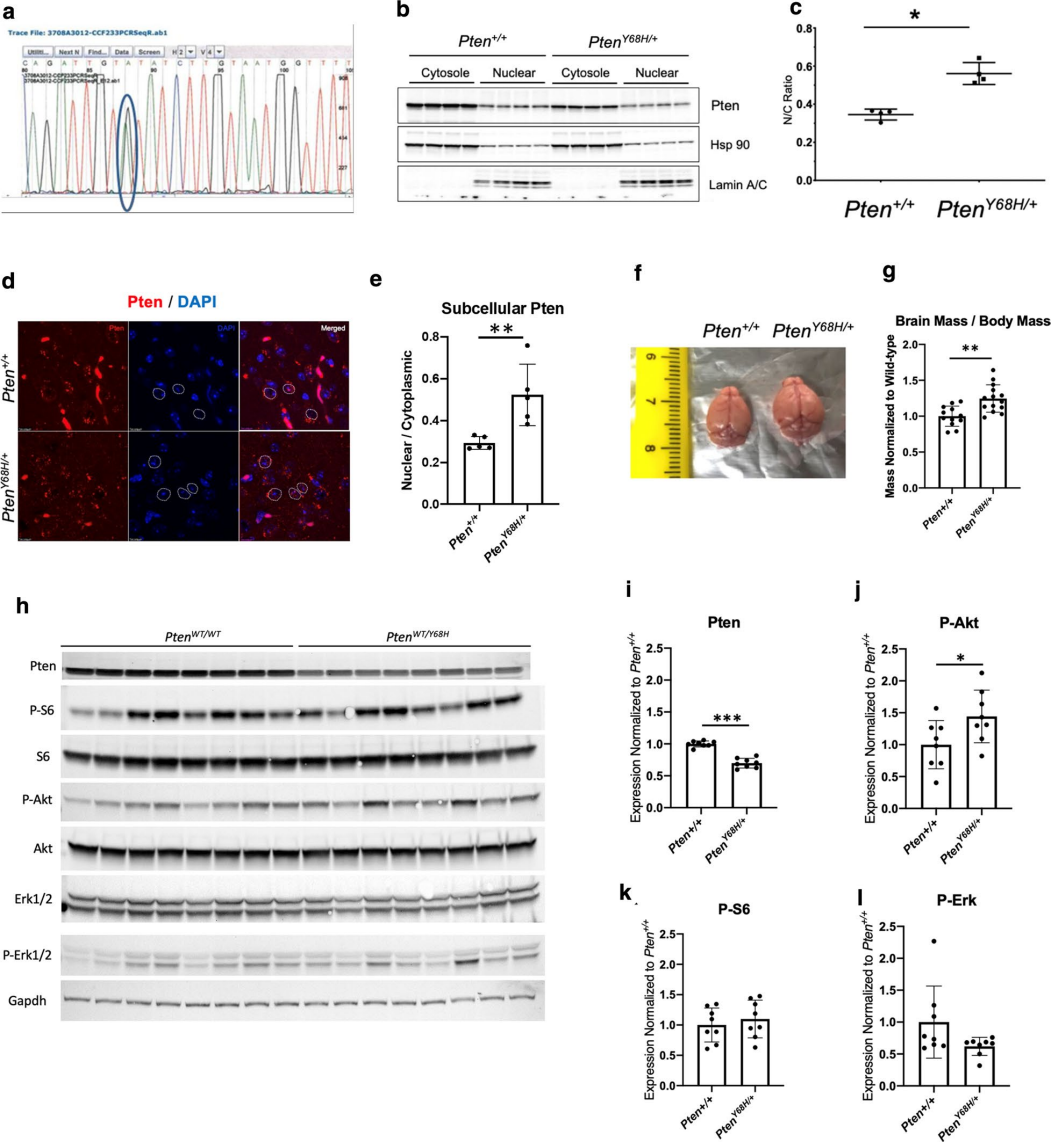
Characterization of gross morphological and Pten localization phenotypes in *Pten*^*Y68H/*+^ mice. **a** Visualization of single missense mutation in exon 3 of mouse *Pten* c.202 T > C, converting tyrosine (Y) residue 68 into histidine (H), which served to generate the germline *Pten*^*Y68H/*+^ mouse model. **b** Nuclear-cytoplasmic fractionation performed on *Pten*^+*/*+^ and *Pten*^*Y68H/*+^ mouse cortical tissue and visualized via Western blot, targeting Pten as well as Hsp90 (cytoplasmic), and Lamin A/C (nuclear) to demonstrate fraction purity. *N* = 4. **c** Quantification of nuclear-cytoplasmic Pten ratio from Western blot in panel b (Median_∆N/CRatio_ = 0.20, 97% CI: 0.14–0.34, *P* = 0.029). **d** Immunofluorescence staining of mouse cortex, visualizing Pten (red), and DAPI (blue) in neurons of *Pten*^+*/*+^ and *Pten*^*Y68H/*+^mice. **e** Quantification of panel d (Median_∆_ = 0.22; 97% CI: 0.089–0.48; *P* = 0.079). **f** Representative gross anatomical image, showing brain volume and morphology of *Pten*^+*/*+^ and *Pten*^*Y68H/*+^mice. **g** Quantification of total brain mass and body weight in grams (g) of *Pten*^+*/*+^and *Pten*^*Y68H/*+^ (median_ΔBrainMass_ = 0.15, 97% CI: 0.090–0.21, *P* < 0.0001). **h** Analysis of downstream Pten signaling in *Pten*^*Y68H/*+^ cortex by Western blot. *N* = 8. **i** Quantification of Pten expression data from panel h (*P* < 0.0002). **j** Quantification of P-Akt levels from panel h (*P* < 0.0002). **k** Quantification of P-S6 expression data from panel h (*P* = 0.50). **l** Quantification of P-Erk1/2 expression data from panel h (*P* = 0.16)

We generated *Pten*^*Y68H/*+^ mice on a C57BL/6 J (Jackson Laboratory, Bar Harbor, Milwaukee) background by introducing one missense mutation into exon three of the mouse *Pten* gene, specifically *Pten* c.202 T>C, via standard cre-lox methodology (Fig. 1a). This mutation targets the sequence analogous to the ATP-binding motif B found in human *PTEN* [32, 33]. Male *Pten*^*Y68H/*+^ mice on a C57BL/6J background (backcrossed for > 10 generations) were bred with female *Pten*^+*/*+^ mice on a C57BL/6 J background newly ordered from Jackson Laboratory. Genotyping was performed on genomic DNA from clipped toes per the Jackson Laboratory protocol using modified PCR primers: Y68H F1, 5′-GTTTCACAGCTGGTTGGAAGG -3′, and Y68H R1, 5′-TGTACCCAGCTCACAGACTTCC-3′. Mice were maintained on a 14:10 light/dark cycle with access to food and water ad libitum. The room temperature was maintained between 18 and 26 °C. Animals were euthanized via CO_2_ asphyxiation followed by cervical dislocation. All experiments were conducted under protocols approved by the Institutional Animal Care and Use Committee (IACUC) at Cleveland Clinic. Additionally, for all experiments described, we utilized both male and female mice for our experiments.

### Western blot analysis

Cortical regions of the brain were isolated, snap-frozen, and stored at − 80 °C. Tissue was thawed on ice and lysed in RIPA buffer (10 mM Tris–Cl [pH 8], 1 mM EDTA, 0.5 mM EGTA, 1% Triton X-100, 0.1% sodium deoxycholate, 0.1% SDS, 140 mM NaCl), containing phosphatase inhibitor #2 (Sigma, St. Louis, Missouri, #P5726-5ML), phosphatase inhibitor #3 (Sigma, #P0044-5ML), and protease inhibitor (Sigma, #P8345-5ML). Lysates were quantified for protein concentration using bicinchoninic acid assay (BCA) assay, equalized to a concentration of 1 μg/μl of protein per sample, and finally 20 μg of protein was loaded to a 4–15% gradient polyacrylamide gel for SDS-PAGE separation. The separated proteins were transferred to a nitrocellulose membrane and blocked overnight at 4 °C in 3% bovine serum albumin (BSA) in 1X Tris-buffered saline, containing 0.2% Tween-20 (TBST). Membranes were then washed with TBST and incubated with experiment-specific primary antibodies diluted in bovine serum albumin (BSA) overnight at 4 °C. The following antibodies were used: PTEN (1:5000, #ABM-2025, Cascade Bioscience, Winchester, Massachusetts), IBA1 (1:500, #019-19741, Wako, Bellwood, Virginia), MBP (1:1000, MAB386, EMD Millipore, Burlington, Massachusetts), PLP (1:1000, ab28486, Abcam, Cambridge, Massachusetts), GAPDH (1:5000, 2118L, Cell Signaling, Danvers, Massachusetts), HSP90 (1:1000, 4874, Cell Signaling), Lamin A/C (1:1000, 2032, Cell Signaling), Beta-actin (1:5000, AM4302, Thermo-Fisher, Waltham, Massachusetts), phospho-AKT Ser473 (1:1000, 9271, Cell Signaling), AKT (1:1000, 4691, Cell Signaling), phospho-ERK1/2 (1:1000, 9101, Cell Signaling), ERK1/2 (1:1000, 9102, Cell Signaling), phosph-S6 (1:1000, 4858S, Cell Signaling), and C1q (1:500, ab71940, Abcam). We removed the primary antibody solution and performed three washes, 10 min per wash, with TBST. Blots were probed with goat anti-mouse secondary antibody IRDye800 (1:20,000, #213965, LI-COR, Lincoln, Nebraska) or goat anti-rabbit IRDye680 (1:20,000, #213971, LI-COR) diluted in BSA, for 2 h at room temperature. The membranes were washed three times, 10 min each in TBST, and imaged using the Odyssey CLx imaging system (LI-COR). Using ImageJ (National Institute of Health, Bethesda, Maryland, 1995), we performed densitometry analysis on these images to quantify protein expression.

### Gene expression analysis by qRT-PCR

Total RNA was extracted from *Pten*^+*/*+^ and *Pten*^*Y68H/*+^ flash frozen cortex using the RNeasy Plus kit (RNeasy Plus kit, # 74136, Qiagen, Germantown, MD) and cDNA synthesis using Maxima First Strand cDNA Synthesis Kit for RT-qPCR (Maxima First Strand cDNA Synthesis Kit for RT-qPCR, #K1642, Thermo-Fisher) according to supplier protocols. We designed primers using UCSC Genome browser mouse GRCm39/mm39 mouse (https://genome.ucsc.edu/) assembly to select exonic regions of interest and selected primers according to standard Primer3 version 0.4.0 (https://bioinfo.ut.ee/primer3-0.4.0/) protocol to select our qRT-PCR primers. qRT-PCR was performed using Power SYBR Green Master Mix (SYBR Green Master Mix, # 4367660, Thermo-Fisher) following standard protocol to prepare samples in a 96-well plate (Fisherbrand 96-Well Semi-Skirted PCR Plates, #14230244, Fisher Scientific, Waltham, MA) and run on QuantStudio™ 3 Real-Time PCR System (QuantStudio™ 3 Real-Time PCR System, # A28567, Thermo-Fisher) as a standard run with cycling program of 10 min at 95 °C followed by 40 cycles of 95 °C for 15 s and 60 °C for 60 s. Primer sequences used and designed for mouse are listed in Additional file 1: Table S1.

### Behavior testing

To assess changes in social behavior, we employed the three-chamber sociability test according to a previously reported protocol [22, 34]. Mice were placed in a center chamber for five minutes and then returned to their original cage. Next, the assessment consisted of a 10-min trial, where the test mouse was returned to the central chamber and given a choice between two identical containers, one chamber containing a mouse, and the other an empty chamber. In order to measure preference for social novelty, the 10-min trial was repeated on the same day later with a familiar mouse in one chamber and a novel mouse in the other. Time spent in each chamber and time spent in close contact with the containers were recorded and quantified using Noldus EthoVision software (Wageningen, Netherlands).

To assess repetitive behavior, we administered the marble burying test to our mice per a previously published protocol [35]. This trial is performed by placing 20 marbles atop clean bedding material and placing the trial mouse into the case for a 30-min session. Upon completion of the trial the number of buried and non-buried marbles was scored.

The open field test was conducted to assess a mouse’s decision to enter or travel through the center of an arena based on the premise that mice experience anxiety in exposed spaces. Tracking software Noldus EthoVision XT (Wageningen, Netherlands) was used to establish set zones in an open field box measuring 48 × 48 × 48 cm (L × W × H) with the center zone measuring 15 × 15 cm. Mice were placed into the center of the box and their location and overall velocity recorded for 10 min using Noldus EthoVision XT (Wageningen, Netherlands). Post-assay mice were placed back into their home cage, and the open field box was cleaned with trifectant. These data were then analyzed to determine the amount of time and frequency the mice entered the center zone of the open field box.

The accelerating rotarod is used to assess sensorimotor coordination and motor learning in rodent models. This was a two-day task in which mice are placed onto a non-rotating rod (3 cm in diameter) and divided into 6 sections that allows 6 mice to be tested at the same time without being able to see each other. The rod is 46 cm above a platform. Each mouse received 4 trials per day with an inter-trial interval of at least 10 min to prevent their performance from being impaired by fatigue. For each trial mice were placed onto the rod and allowed to balance themselves. When all mice were balanced on the rod, rod rotation was initiated and slowly accelerated from 4 to 40 revolutions per-minute (rpm) over a 5-min period. Latency to stay on the rod was assessed using an infrared beam below the rod that when triggered would record a mouse’s latency to fall. After the 4 trials were completed, mice were returned to their home cage and the floor and rod were cleaned with trifectant. Each mouse received a total of 8 trials over a two-day period (4 trial/day with and intertrial interval of at least 10 min).

Additionally, to determine whether there was altered communication of mutant mice, we recorded ultrasonic vocalizations (USVs) of pups isolated from their mothers and litter mates. On postnatal day 4 (P4) mice are labeled with a black marker for identification on their backs and separated from mother and littermates. Separated pups were placed into a clean weigh-boat inside a sound-attenuating Styrofoam box from which USVs were recorded with ultrasound microphone Avisoft UltraSoundGate condenser microphone capsule CM16, Avisoft Bioacoustics (Glienicke/Nordbahn, Germany) for 5 min. After the recording pups were returned to their dam and litter, the number of calls was counted and analyzed using Avisoft SASLab Pro software (Glienicke/Nordbahn, Germany). This test was conducted as a blinded study, and mouse genotype was unknown until P8 post toe clip and genotyping.

### Primary microglia cell culture

Mixed glia were obtained by trypsinization of postnatal day 2 (P2) cortices followed by plating on poly-d-lysine-coated T-75 culture flasks. Mixed glia cultures were maintained in DMEM (Lerner Research Institute Media Core, Cleveland, OH) with 10% FBS and 1% Penicillin and Streptomycin (Pen/Strep). Once the mixed glia cultures reached confluency at approximately DIV 10, they were agitated for 1 h at 125 RPM. At this point, the supernatant was removed and spun down at 1200 RMP in order to isolate primary microglia. Isolated microglia were resuspended in DMEM with 10% FBS and 1% Pen/Strep and seeded on poly-d-lysine-coated glass cover slips subsequently used for immunofluorescence staining and phagocytic assays at DIV 3 post-shaking.

### Phagocytosis assay

We plated primary microglia at a density of 1 × 10^5^ in a 12-well dish with PDL-coated coverslips for 48 h in a 37 °C cell incubator with 5% CO_2_ and 100% humidity. Next, we blocked 1-μm fluorescent beads (Sigma-Aldrich, #L1030) in FBS for 1 h at 37 °C at a ratio of 1:5 v/v. Florescent beads were diluted with DMEM to reach a final concentration of 0.01% (v/v). Microglial culture media were replaced with 250 μl DMEM containing beads and incubated for 1 h at 37 °C in a cell incubator. Cultures were washed thoroughly five times with ice-cold PBS (Lerner Research Institute Media Core) and fixed in ice-cold methanol prior to immunofluorescent staining for IBA1 (1:500, #019-19741, Wako).

### Immunofluorescence staining of brain tissue

Mice were euthanized and perfused with approximately 50 ml of 1X PBS. Brain tissue was then extracted and fixed in 4% PFA (pH = 7) for 24 h at 4 °C. Brains were then washed three times with PBS and cryoprotected in 30% sucrose dissolved in PBS for 94 h at 4 °C. Frozen brain sections were cut coronally to a width 10 μm on a cryostat and mounted on polarized glass slides (Fisherbrand Superfrost Plus microscope slides, #12-550-15, Fisher Scientific). OCT was removed by washing slides in PBS for 10 min, and tissue was permeabilized with 3% Triton-X dissolved in PBS for 10 min. Slides were next washed three times for five minutes each in PBS and probed with experiment-specific primary antibodies: IBA1 (1:500, #019-19741, Wako), PLP (1:1000, ab28486, Abcam), NeuN (1:250, MAB377, EMD Millipore), OLIG2 (1:250, ab9610, Abcam), S100B (1:200, ab52642, Abcam), GFAP (1:250, sc-33673, Santa Cruz), OXT (1:250, ab212193, Abcam), PTEN (1:5000, #ABM-2025, Cascade Bioscience) and incubated overnight at 4 °C. The following day, slides were washed with PBS for three times five minutes each. This was followed by incubation with secondary antibody for 2 h: goat anti-mouse Alexa Fluor 568 (1:2000, #A11031, Thermo-Fisher) and goat anti-rabbit Alexa Fluor 488 (1:2000, #A11008, Thermo-Fisher). Post incubation, slides were washed and mounted in Vectashield medium with DAPI (Vector Laboratories, Burlingame, CA), coverslipped, and sealed with nail polish.

### In vitro immunofluorescence staining

We cultured primary microglia on poly-D-lysine (PDL)-coated cover slips until DIV 14. Microglia were washed with ice-cold PBS and fixed in ice-cold methanol for two minutes. This was followed by three washes for five minutes each with ice-cold PBS. We then permeabilized the microglia with 0.03% Triton X-100 dissolved in PBS for four minutes. Next, cells were blocked with 10% normal goat serum for 1 h at room temperature, followed by incubation with primary antibody IBA1 (1:500, #019-19741, Wako), CX3CR1 (1:250, #14-6093-81, Thermo-Fisher) TREM2 (1:250, #76765S, Cell Signaling) diluted in 10% normal goat serum in PBS. Cells were then incubated in primary antibody overnight at 4 °C. The following day cells were washed with PBS three times for five minutes and secondary was added, goat anti-mouse Alexa Fluor 568 secondary antibody (1:2000, #A11031, Thermo-Fisher) diluted in 10% normal goat serum in PBS. The cells were incubated in secondary antibody for 2 h at room temperature, washed with PBS three times for five minutes, and coverslipped with Vectashield medium with DAPI (Vector Laboratories).

### Immunofluorescence quantification

We captured images of brain sections and primary microglia as confocal images using a Leica TCS-SP8-AOBS inverted confocal microscope (Leica Microsystems, GmbH, Wetzlar, Germany). Brain sections and microglia cultures were imaged with a minimum of *N* = 3 biological replicates. ImageJ software was used to measure area and intensity of the stain and calculated integrated density of brain images. Additionally, ImageJ was used to measure area of stain per microglia in vivo to assess morphological changes.

### Transcriptomic data analysis

We isolated total RNA from the cortex of eight *Pten*^*Y68H/*+^ mice and seven *Pten*^+*/*+^ mice. Aliquots of roughly 60 ng/μL total RNA (average RIN score = 9.1; Additional file 1: Table S2) were prepared (TruSeq Stranded Total RNA—RiboZero Gold, Illumina, San Diego, CA) and then sequenced using an Illumina NOVA-Seq. The resulting Fastq sequences were subject to standard processing and quality control (QC) evaluation, using MultiQC v1.9 (https://multiqc.info/). Then, we performed an alignment to the mouse reference genome (mm10) using Spliced Transcripts Alignment to a Reference (STAR) 2.7.5 (https://github.com/alexdobin/STAR) [36–38] and repeated a quality control evaluation using MultiQC v1.9. One *Pten*^+*/*+^ sample and three *Pten*^*Y68H/*+^ samples were discarded due to a high proportion of repetitive sequences and generally poor alignment statistics (Additional file 1: Fig S1). Additionally, we used Salmon 1.8.0 (https://bioconductor.org/packages/release/workflows/html/rnaseqDTU.html) as an alternative method to count reads mapping to a present index of known cDNA transcripts. Subsequently, we performed DeSeq2 1.28.1 on STAR-aligned counts and Salmon-produced counts to assess differential expression (DE). These two methods were used to ensure concordance between both approaches. Genes experiencing DE were analyzed in RStudio 1.2.5001 using R 4.0.0 to construct volcano plots and heatmaps. Generally, a *p *value (*P* < 0.05), fold change (Log_2_(Fold Change) ≥ 1.0 or (Log_2_(Fold Change) ≤ − 1.0), and count (RPKM > 10) thresholds were used for these analyses. In order to assess the biological impact of the DE results, we used STRING (https://string-db.org/) and Ingenuity Pathway Analysis (Qiagen, Redwood City, California) software.

### Glial transcriptomic analysis with NanoString panel

To supplement our cortical total RNA sequencing and gain some greater insight into glia-specific molecular profiles, we utilized NanoString nCounter® Glial Profiling Panel (https://www.nanostring.com/products/ncounter-assays-panels/neuroscience/glial-profiling/) on P40 cortical RNA extracted from *Pten*^*Y68H/*+^ and *Pten*^+*/*+^ littermate controls equal parts male and female (*N* = 8), and then quantified differential expression. This approach reports on nearly 800 glial-related genes. NanoString is a direct multiplexed method that measures gene expression using color-coded probes. The original descriptive methods for this approach are found in Geiss et al., 2008 [39].

### Statistical analysis

We analyzed normally distributed data using a one-way analysis of variance (ANOVA) or Student’s t test, where appropriate (GraphPad Prism 8). After performing a one-way ANOVA (F), we performed a post hoc Tukey–Kramer analysis. When data were not normally distributed, we performed nonparametric analyses including Mann–Whitney U and Kruskal–Wallis tests (H), where appropriate (GraphPad Prism 8). *P* values that are less than 0.05 were considered statistically significant.

## Results

### ***Pten***^***Y68H/***+^ mice exhibit increased nuclear Pten localization and increased brain mass

We originally observed the *PTEN*^*Y68H*^ mutation in PHTS individuals diagnosed with ASD and found that this particular mutation was sufficient to disrupt the subcellular partitioning of PTEN resulting in relatively predominant nuclear localization [33]. Furthermore, we generated the *Pten*^*Y68H/*+^ mouse model by introducing a single missense mutation into exon three of mouse *Pten* (i.e., *Pten* c.202 T > C), thus converting tyrosine residue 68 into histidine (Fig. 1a). To assess the subcellular localization of Pten in our *Pten*^*Y68H/*+^ mouse, we performed nuclear-cytoplasmic fractionation of cortical tissue and assessed protein localization via Western blot (Fig. 1b). We observed decreased Pten in the cytoplasmic fraction of *Pten*^*Y68H/*+^ hemibrain relative to *Pten*^+*/*+^ (*N* = 4 mouse/genotype, Fig. 1b). Additionally, quantitative assessment showed that the ratio of nuclear-to-cytoplasmic Pten is increased in the *Pten*^*Y68H/*+^ mice compared to *Pten*^+*/*+^ (Median_∆N/CRatio_ = 0.20; 97% CI: 0.15–0.34; *P* = 0.029; Fig. 1c). Next, we performed immunofluorescence staining for Pten in the brains of six-month-old *Pten*^*Y68H/*+^ mice, which also shows predominantly nuclear localization of Pten (Median_∆N/CRatio_ = 0.22; 97% CI: 0.089–0.48; *P* = 0.079; Fig. 1d, e). These observations are consistent with our Western data on subcellular fractionation (Fig. 1b), showing enrichment of nuclear Pten localization relative to cytoplasmic Pten in the *Pten*^*Y68H/*+^ brain (Fig. 1d).

Macrocephaly is a hallmark of PHTS individuals, and ASD in the context of germline *PTEN* mutation is always accompanied by macrocephaly [28, 40, 41]; therefore, to determine whether a similar overgrowth phenotype exists in *Pten*^*Y68H/*+^ mice, we performed a gross examination of *Pten*^*Y68H/*+^ brains at P40. We found a significant increase in brain mass as measured in grams in *Pten*^*Y68H/*+^ mice compared to *Pten*^+*/*+^ littermate controls normalized to individual mouse body mass (Median_ΔBrainMass/BodyMass_ = 0.24; 97% CI: 0.089–0.38; *P* < 0.001; Fig. 1f, g).

### ***Pten***^***Y68H/***+^ mice show decreased expression of Pten and increased phosphorylation of Akt

To evaluate overall Pten expression and downstream signaling in *Pten*^*Y68H/*+^ mice, we performed Western blot analysis on cortical lysates isolated from *Pten*^*Y68H/*+^ mice and *Pten*^+*/*+^ littermate controls at six weeks-of-age. We found a significant decreased in overall Pten expression in the cortical lysates of *Pten*^*Y68H/*+^ mice (Median_ΔPten_ = − 0.31; 95% CI: − 0.38 to − 0.23; *P* < 0.0002) (Fig. 1h, i). In addition, we blotted for the canonical downstream effectors of Pi3k/Akt/mTor signaling, finding significantly increased P-Akt levels in the cortices of *Pten*^*Y68h/*+^ mice (Median_ΔP-Akt/TotalAkt_ = − 0.38; 95% CI: 0.038–0.89; *P* < 0.0002) with no changes in P-S6 or P-Erk1/2 (Fig. 1h, j, k, l).

### ***Pten***^***Y68H/***+^ mutant mice exhibit behavioral deficits

**Fig. 2:**
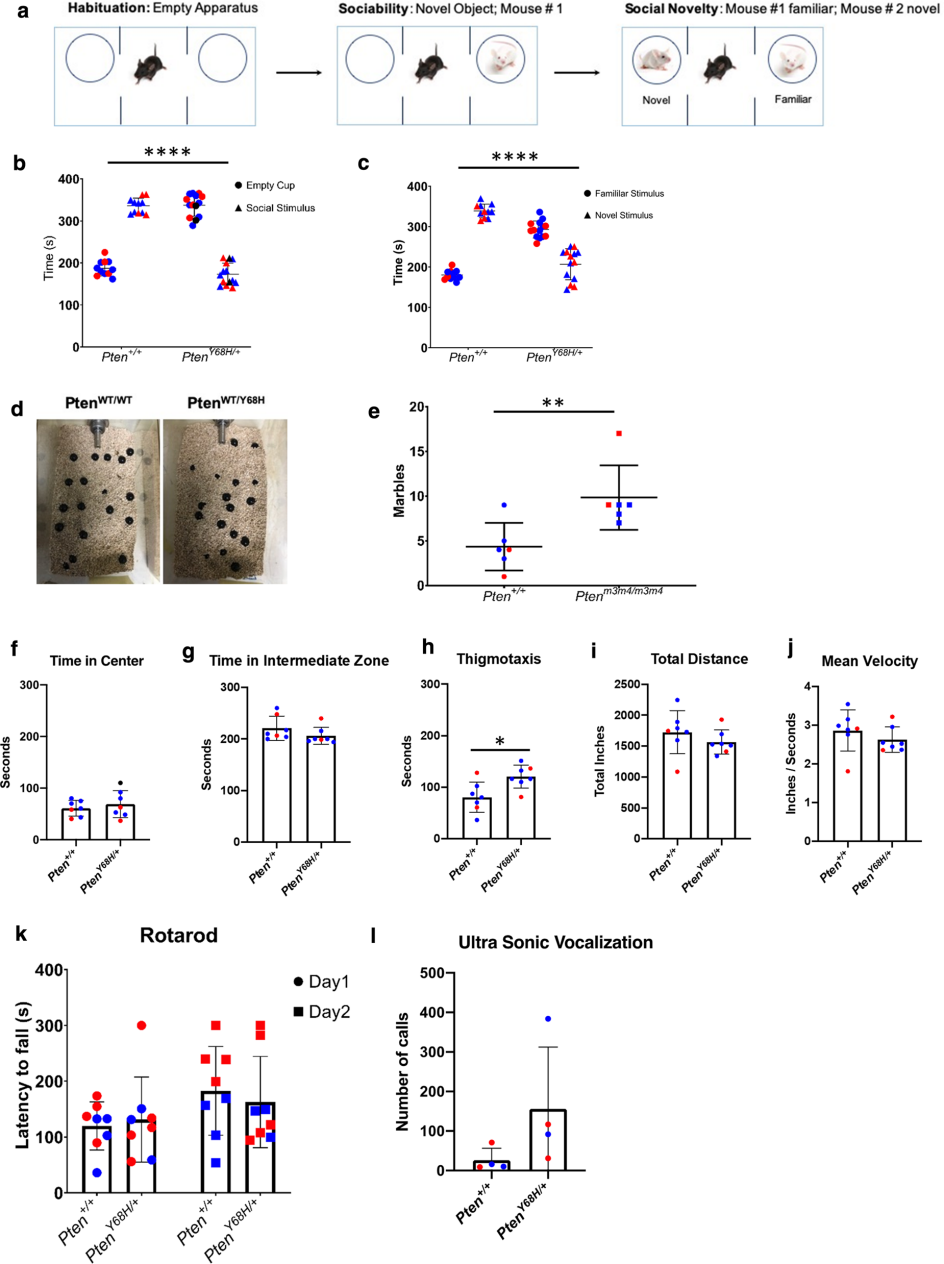
Behavioral phenotyping of *Pten*^*Y68H/*+^ mice. **a** Schematic of the experimental design for the three-chamber sociability and social novelty tests as they were performed on six-week-old *Pten*^+*/*+^ and *Pten*^*Y68H/*+^ mice. **b** Quantification of the three-chamber sociability test, showing time spent in empty chamber or in chamber containing the social target (*P* < 0.0001; N_+/+_  = 13; N_Y68H/+_  = 10). **c** Quantification of the three-chamber social novelty test performed with six-week-old *Pten*^+*/*+^ and *Pten*^*Y68H/*+^ mice, showing time spent in chamber containing familiar or novel social target (*P* < 0.0001; N_+/+_  = 13; N_Y68H/+_  = 10). **d** Representative image of marble burring test results for *Pten*^+*/*+^ and *Pten*^*Y68H/*+^ mice. **e** Quantification of marble burying test, showing mean marbles buried between *Pten*^+*/*+^ and *Pten*^*Y68H/*+^ mice (Median_ΔMarbles_ = 4.68; 97% CI: 2.12–7.23; *P* = 0.002). **f** From open field test (*N* = 6), the amount of time (s) mice spent in center of field (*P* = 0.65). **g** Time in intermediate zone (s) assessed by open field test (*P* = 0.078). **h** Time in thigmotaxis (s) as assessed by open field test (*P* = 0.018). **i** Total distance traveled during open field testing (*P* = 0.16). **j** mean velocity of mice during open field testing (*P* = 0.26). **k** Rotarod testing experiment (*N* = 8; *P*_genotype_ = 0.90; *P*_learning_ = 0.01). **l** Ultrasonic vocalization testing (*N* = 4; *P* = 0.057). Datapoints are colored by mouse sex. Males in blue. Females in red. *P* value key: *P < 0.05, **P < 0.01, ****P < 0.0001

Next, we sought to examine the possibility that nuclear-predominant Pten expression in the central nervous system (CNS) may alter social behavior. Several studies have demonstrated that Pten loss, whether constitutional or conditional to the CNS, can have deleterious consequences on social behavior, anxiety, learning, memory, and/or repetitive behavior, phenotypes that are associated with ASD in humans [15, 28]. In order to assess whether there were any changes in sociability at six-week-old *Pten*^*Y68H/*+^ mice compared to *Pten*^+*/*+^ littermate controls, we employed the three-chamber test (Fig. 2a). We found that *Pten*^*Y68H/*+^ mice spent less time in the chamber containing the social target than the empty chamber (*P* < 0.0001; N_+/+_  = 13; N_Y68H/+_  = 10; Fig. 2b). Then, utilizing the same three-chamber test model, we assessed changes in preference for social novelty by placing a familiar mouse in one chamber and a novel social target in the other. We found a significant shift for *Pten*^*Y68H/*+^ mice toward a reduced preference for a novel versus familiar social target compared to *Pten*^+*/*+^ littermate controls (*P* < 0.0001; N_+/+_  = 13; N_Y68H/+_  = 10; Fig. 2c). In order to assess repetitive behavior, since it is defined as one of the two core behavioral domains of ASD [42], we performed the marble burying test with six-week-old *Pten*^*Y68H/*+^ mice. We found that the cages of *Pten*^*Y68H/*+^ mice had more marbles buried compared to *Pten*^+*/*+^ controls (Median_ΔMarbles_ = 4.68; 97% CI: 2.12–7.23; *P* = 0.002) and increased displacement of the marbles from their original positions compared to the cages of wildtype mice as assessed by visual inspection (Fig. 2d, e). The social deficits observed in *Pten*^*Y68H/*+^ mice roughly align with the DSM-V behavioral domains for ASD diagnosis: social impairment and restrictive and repetitive interests or behaviors.

To expand our behavioral phenotyping and assess anxiety in our model, we performed open field testing. The open field test helps determine whether *Pten*^*Y68H/*+^ mice have altered interests in exploration and the extent they experience anxiety. We found no significant changes in time spent in the center, and intermediate zone of the open field arena (Fig. 2f, g); however, we did find that *Pten*^*Y68H/*+^ mice preferred to travel and stay along the walls of the open field arena, which indicates increased thigmotaxis (Median_Δ_ = 44.6; 97% CI: 7.97–73.7; *P* = 0.018; Fig. 2h). Increased thigmotaxis is a potential indicator of anxiety. Further analysis of the *Pten*^*Y68H/*+^ total distance traveled and velocity did not show any significant differences compared to *Pten*^+*/*+^ mice, but there was a trend toward decreased distance traveled and exploratory movement in *Pten*^*Y68H/*+^ mice relative to controls (Fig. 2i, j). The open field test suggests that *Pten*^*Y68H/*+^ mice may experience more anxiety than their wildtype counterparts.

We also sought to assess motor coordination and neuromuscular learning via rotarod testing. We found no difference in latency to fall between *Pten*^*Y68H/*+^ and *Pten*^+*/*+^ mice, and both sets of mice improved their latency to fall upon successive testing but also showed no significant differences in motor learning (Fig. 2k). It appears there are not deficits in motor coordination in *Pten*^*Y68H/*+^ mice. The lack of motor coordination deficit lends support to the validity of the social deficits observed in the 3-chamber and open field tests.

To learn more about the social behavioral deficits in *Pten*^*Y68H/*+^ mice, we assessed the ultrasonic vocalizations (USVs) of *Pten*^*Y68H/*+^ pups separated from their mothers to determine whether there were any changes in call frequency. We found a near-significant trend toward an increase in call frequency from *Pten*^*Y68H/*+^ pups separated from mothers compared to littermate controls (*P* = 0.057), suggesting greater anxiety in *Pten*^*Y68H/*+^ mice (Fig. 2l). All together these behavioral data demonstrate that *Pten*^*Y68H/*+^ mice exhibit decreased sociability, decreased interest in social novelty, increased repetitive behavior, and increased indices of anxiety. This appears largely consistent with autism-like phenotypes.

### Microglial activation and increased expression of complement and neuroinflammatory proteins in the brains of ***Pten***^***Y68H/***+^ mice

It has already been well established that disrupted Pten expression in mice can lead to increased cellular proliferation, white matter abnormality, astrogliosis, and microglial activation in vivo [15, 22, 29, 30]. Therefore, we sought to determine whether there were any clear cellular pathologies in the brains of *Pten*^*Y68H/*+^ mice, using immunofluorescence staining, qRT-PCR, and Western blotting for markers specific to neurons, oligodendrocytes, astrocytes, and microglia. To our surprise we did not observe any significant abnormalities in gross white matter nor in the populations of oligodendrocytes, astrocytes, or neurons with respect to proliferation or activity the brains of six-month-old *Pten*^*Y68H/*+^ mice (Additional file 1: Fig S2A-H); however, there was a trend showing a slight decline in cell populations of oligodendrocytes and astrocytes in *Pten*^*Y68H/*+^ mice compared to *Pten*^+*/*+^ controls (Additional file 1: Fig S2B-D). Given the advanced age of these mice relative to our other timepoints, we felt it was unlikely there were glial pathologies at an earlier timepoint. We confirmed this by assessing the glial population at P40 too (Additional file 1: Fig S3).

**Fig. 3:**
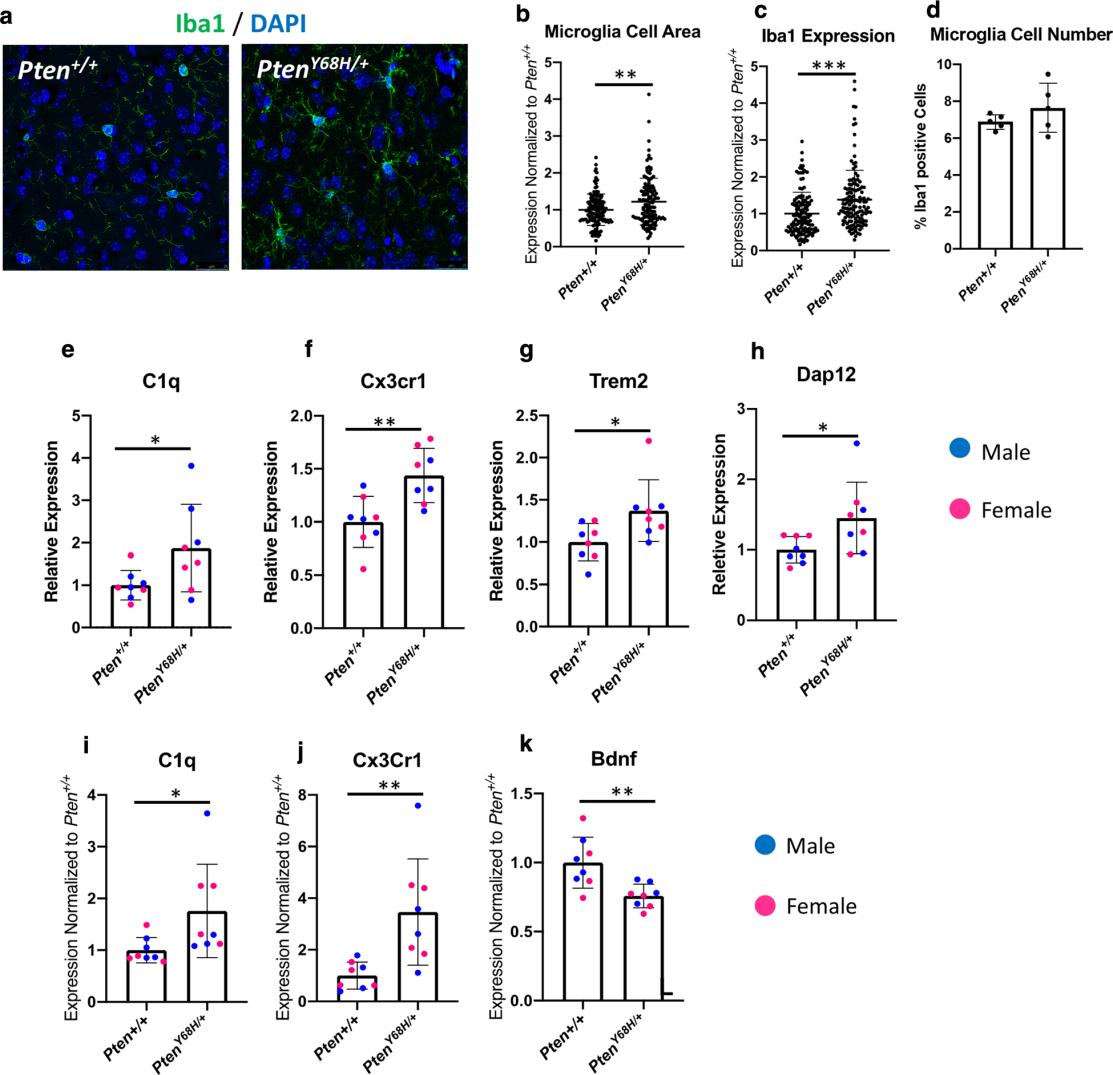
Evidence for a microglial pathology in the *Pten*^*Y68H/*+^ cortex. **a** Representative immunofluorescence staining of six-week-old *Pten*^+*/*+^ and *Pten*^*Y68H/*+^ cortical microglia with Iba1 (green) and DAPI (blue). *N* = 3. **b** Quantification of individual microglia area from data represented in panel a (*P* = 0.007) **c** Iba1 expression assessed from IF staining from panel a experiments (*P* = 0.001). **d** Total Iba1-positive cells normalized to total DAPI-positive cells from panel a experiments (*P* = 0.42). **e–h** Relative expression assessed on cortical tissue from six-week-old *Pten*^+*/*+^ and *Pten*^*Y68H/*+^ mice (*N* = 7) via qRT-PCR for *C1q* (*P* = 0.04; **e**), *Cx3cr1* (*P* = 0.003; **f**), *Trem2* (*P* = 0.02; **g**), and *Dap12* (*P* = 0.03; **h**). **i**-**j** Western analysis on cortical tissue from six-week-old *Pten*^+*/*+^ and *Pten*^*Y68H/*+^ mice (*N* = 8) for C1q (*P* = 0.01; **i**), Cx3cr1 (*P* = 0.001: **j**), and Bdnf (*P* = 0.004; **k**). *P* value key: *P < 0.05, **P < 0.01, ***P < 0.001

During our comprehensive cellular phenotyping of the cortex of six-month-old *Pten*^*Y68H/*+^ mice, we noticed a pattern of microglial activation. Thus, we sought to repeat this observation at our earlier six-week timepoint given that our behavioral data are obtained there. We stained microglia for Iba1 in the cortex of P40 *Pten*^*Y68H/*+^ mice and observed increased Iba1-positive cells and morphological changes indicative of microglial activation, such as increased cell area and Iba1 expression (Fig. 3a, b). We quantified the cell area of individual microglia in *Pten*^*Y68H/*+^ in vivo and found a significant increase in the cell area of these microglia (Mean_∆CellArea_ = 0.22; 95% CI: 0.04–0.28; *P* = 0.007; Fig. 3b). Moreover, we measured the integrated density of the Iba1 stain and found it to be significantly increased in *Pten*^*Y68H/*+^ compared to *Pten*^+*/*+^ microglia (Mean_∆IntDensity_ = 0.33; 95% CI: 0.19–0.45; *P* = 0.0001; Fig. 3c). Finally, to confirm these changes were not the result of increased microglial number, we quantified the microglia numbers of mice assayed for microglia area and Iba1 expression (*N* = 5) and found no significant change in *Pten*^*Y68H/*+^ mice compared to *Pten*^+*/*+^ (Fig. 3d).

Next, we sought to validate our earlier observations and determine whether there was increased expression of key complement and neurodegenerative genes in the cortex of P40 *Pten*^*Y68H/*+^ mice using qRT-PCR. We found significantly increased expression in *C1q* (Mean_∆RelativeExpression_ = 0.87; 95% CI: 0.05–1.70; *P* = 0.04; Fig. 3e), *Cx3cr1* (Mean_∆RelativeExpression_ = 0.44; 95% CI: 0.17–0.70; *P* = 0.003; Fig. 3f), *Trem2* (Mean_∆RelativeExpression_ = 0.37; 95% CI: 0.05–0.69; *P* = 0.02; Fig. 3g), and *Dap12* (Mean_∆RelativeExpression_ = 0.45; 95% CI: 0.04–0.86; *P* = 0.03; Fig. 3e). We did not see any significant changes in complement genes *C3, C3ar1, Csf1r,* and *Itgam* or genes essential for fractalkine and immune signaling such as *Cx3cl1, Ccl2, Igf1, Adam10, Adam17, Tlr4, Il4,* and *Il10*, excepting *Itbg2* (Additional file 1: Fig. S4).

Next, we examined protein expression of known microglia or neurotrophic makers: C1q, Cx3cr1, and Bdnf via Western blot. We found significantly increased expression of C1q (Mean_∆Expression_ = 0.76; 95% CI: 0.19–1.38; *P* = 0.01; Fig. 3i), Cx3cr1 (Mean_∆Expression_ = 3.4; 95% CI: 0.72–3.87; *P* = 0.001; Fig. 3j), and Bdnf (Mean_∆Expression_ = -0.24; 95% CI: − 0.04 to − 0.09; *P* = 0.004; Fig. 3e). These data suggest not only that microglia are activated and have a pro-inflammatory molecular signature, but also that they may participate in increase phagocytosis given the increased expression of complement. Together, these data indicate that the *Pten*^*Y68H*^ mutation appears to leave astrocyte and oligodendrocyte populations unaffected, while contributing to a microglial pathology in vivo. This phenotype is also stable throughout the early and later life of the model.

### Primary ***Pten***^***Y68H/***+^ microglia express increased C1q and exhibit increased phagocytic ability in vitro

**Fig. 4:**
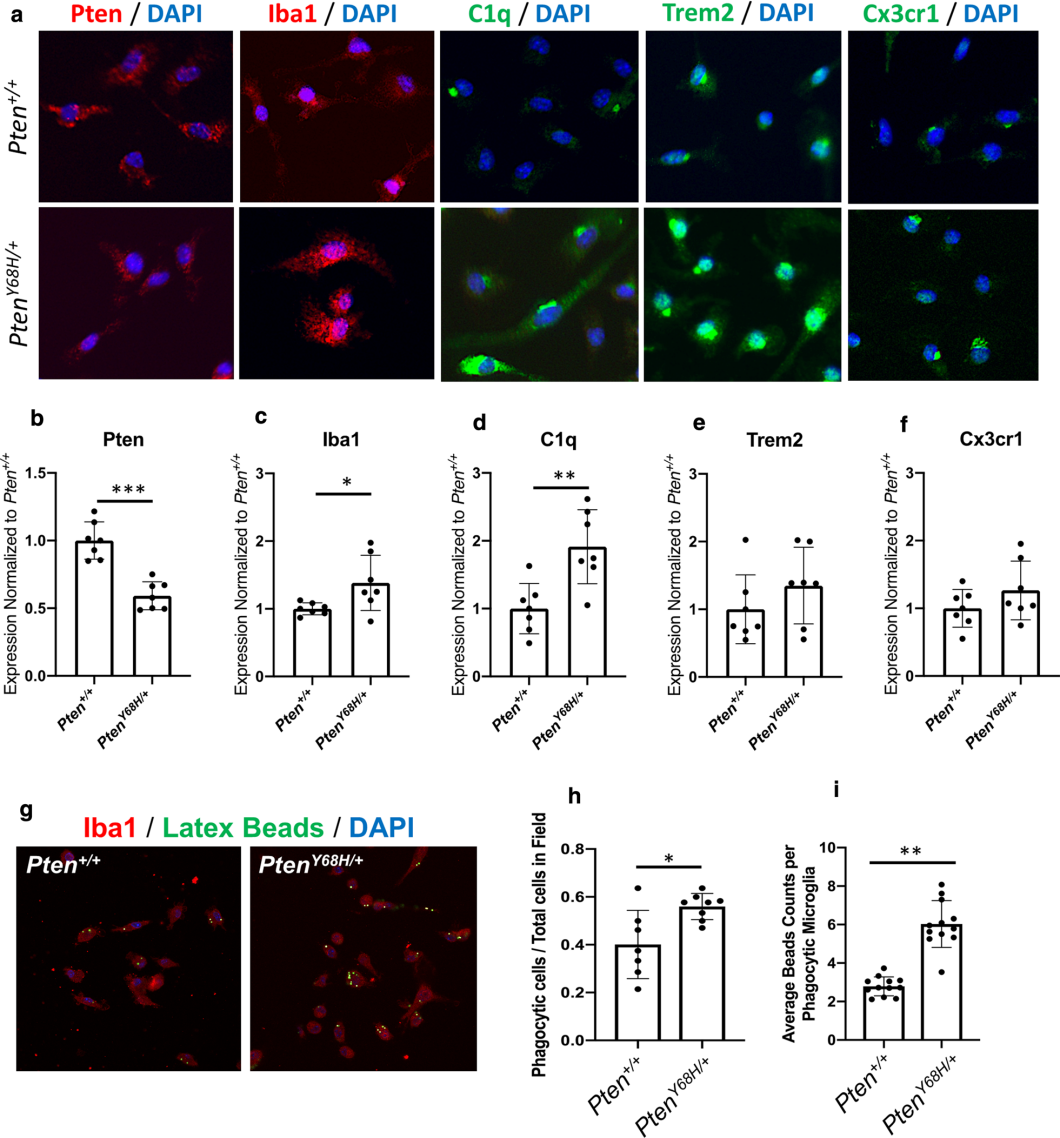
In vitro validation of *Pten*^*Y68H*^ microglia pathology. **a** Representative immunofluorescence staining of *Pten*^+*/*+^ and *Pten*^*Y68H/*+^ primary microglia with Pten (red), Iba1 (red), C1q (green), Trem2 (green), Cx3cr1 (green), and DAPI (blue). *N* = 7. **b-e** Quantification of IF staining in panel a for Pten (*P* = 0.0006; **b)**, Iba1 (*P* = 0.038; **c**), C1q (*P* = 0.004; **d**), Trem2 (*P* = NS; **e**), and Cx3cr1 (*P* = NS; **f**). **g** Representative image of phagocytosis assay comparing *Pten*^+*/*+^ and *Pten*^*Y68H/*+^ microglia. **h** Quantification of panel g for proportion of active phagocytic microglia over total microglia. *P* = 0.01 **i** Quantification of panel g of average bead counts per phagocytic microglia. *P* = 0.005. *P* value key: *P < 0.05, **P < 0.01, ***P < 0.001

To evaluate the cellular origins (i.e., cell autonomous or cell non-autonomous) of the observed microglia pathology, we cultured primary microglia isolated from *Pten*^*Y68H/*+^ and *Pten*^+*/*+^ mice. We found Pten expression to be significantly decreased overall in *Pten*^*Y68H/*+^ microglia compared to *Pten*^+*/*+^ microglia (Mean_∆Expression_ = -0.42; 95% CI: − 0.55 to − 0.26; *P* = 0.0006; Fig. 4a, b) with significantly increased expression in Iba1 (Mean_∆Expression_ = 0.25; 95% CI: 0.04–0.89; *P* = 0.038; Fig. 4a, C) and C1q (Mean_∆Expression_ = 0.74; 95% CI: 0.36–1.55; *P* = 0.004; Fig. 4a, d). However, we did not see a statistically significant increase in Trem2 or Cx3cr1 as we did in vivo (Fig. 4a, e, f). This suggests the dysregulation of Iba1 and C1q may be of cell autonomous origins, while dysregulation of Trem2 and Cx3cr1 may require some stimuli or interaction.

To assess the phagocytosis activity of cultured *Pten*^*Y68H/*+^ microglia, we performed a phagocytosis assay and found the total number of *Pten*^*Y68H/*+^ phagocytic microglia was significantly higher than the number of phagocytic microglia isolated from *Pten*^+*/*+^ littermate controls (Mean_ΔPhagocyticAbility_ = 0.16; 95% CI: 0.040–0.27; *P* = 0.01; Fig. 4g, h). In addition, phagocytic *Pten*^*Y68H/*+^ microglia were able to engulf more fluorescent beads compared to *Pten*^+*/*+^ littermate controls (Mean_ΔPhagocyticEfficiency_ = 3.2; 95% CI: 2.5–4.0; *P* = 0.005; Fig. 4g, i). These data demonstrate that *Pten*^*Y68H/*+^ microglia are likely subject to largely cell autonomous mechanisms of dysregulation. The lesion in Pten provokes aberrant microglial activation and increases in engulfment activity, which may have effects on the synaptic architecture of the *Pten*^*Y68H/*+^ brain.

### Transcriptomic characterization of the ***Pten***^***Y68H/***+^ cortex

**Fig. 5:**
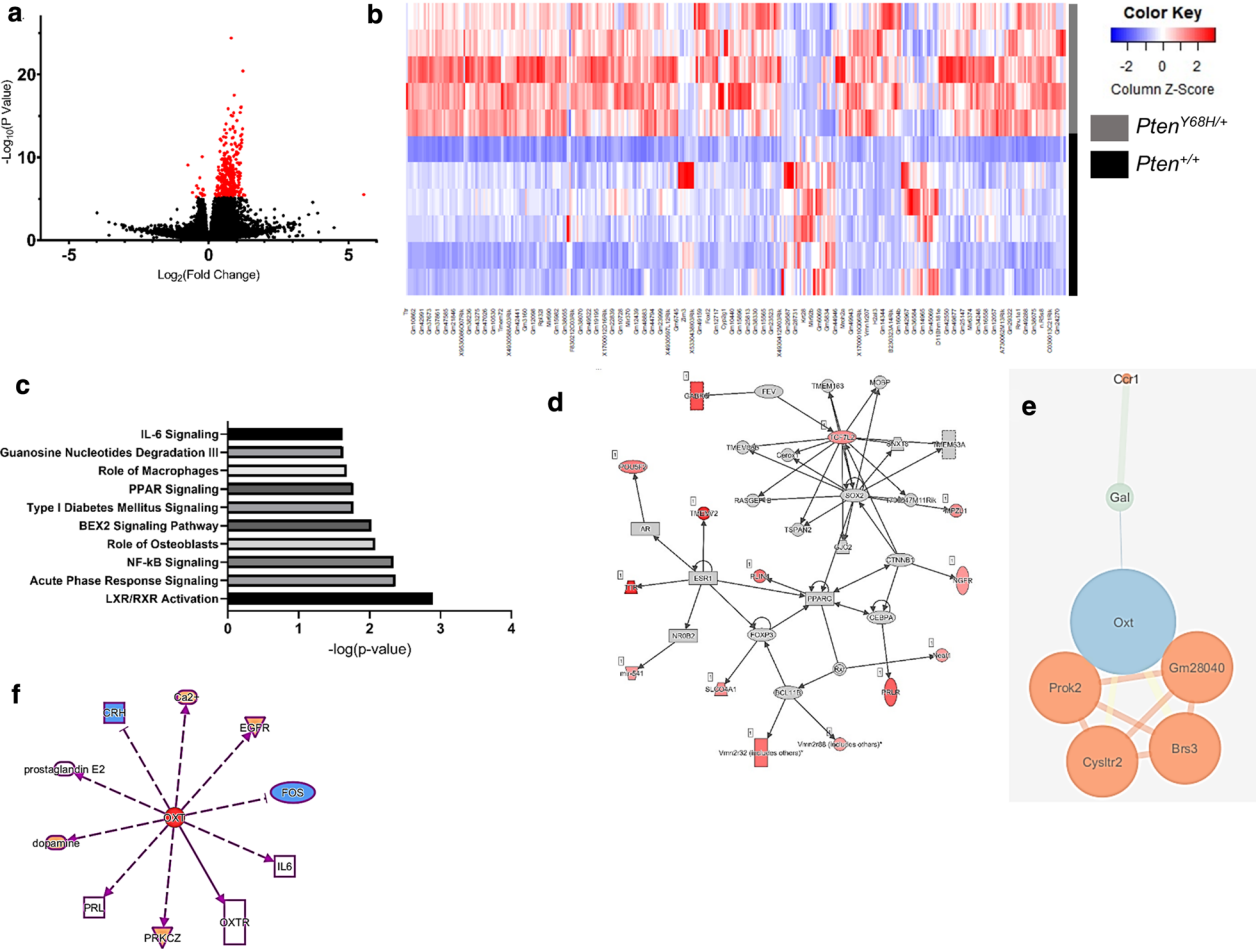
Transcriptomic survey of *Pten*^*Y68H/*+^ cortex identifies broad changes in expression, affecting PTEN signaling and neurological processes, including oxytocin overexpression. **a** Volcano plot highlighting genes showing highly significant changes in differential expression (DE) in red (*P* < 0.0001; N_WT_ = 6; N_Y68H_ = 5). **b** Heatmap of DE genes (threshold: *P* < 0.001) clustered by column (i.e., by expression pattern within a gene). **c** Top “Canonical Pathways” identified by Ingenuity Pathway Analysis (IPA) from DE gene list input. **d** Top identified IPA network from input DE gene list. The network is organized hierarchically. Red = overexpression; green = underexpression; solid line = direct relationship; hashed line = indirect relationship; arrow direction = direction of relationship. **e** STRING analysis of subnetwork of DE genes displaying the relationship between overexpressed Oxt and other DE genes (i.e., interactome). Node size trends with degree connectivity. Node color trends with betweenness centrality (i.e., cooler colors indicated higher betweenness centrality). Thickness of edge trends with the confidence in the biological relationship. **f** An oxytocin regulatory network constructed using IPA’s Grow and Molecule Activity Predictor (MAP) tools. Blue = predicted inactivation; orange = predicted activation; solid line = direct relationship; hashed line = indirect relationship; arrow direction = direction of relationship

Given the surprising lack of disruption of canonical signaling downstream of Pten in the context of the striking behavioral and cellular findings, we performed a transcriptomic survey of the cortex of young adult (six-week-old or P40), male mice (N_+/+_  = 6; N_Y68H/+_  = 5). RNA-sequencing analysis of cortical RNA identified 332 differentially expressed genes (threshold: *P* < 0.05; Log_2_(Fold Change) ≥ 1.0 or Log_2_(Fold Change) ≤ -1.0; Additional file 2), which are summarized in a volcano plot (Fig. 5a). The volcano plot also illustrates askew toward overexpression with relatively fewer under expressed genes being observed. Moreover, the changes in gene expression are visualized in a heatmap (threshold: *P* < 0.001), showing a clear separation between genotypes with a general pattern of increased expression in the heterozygous mutant and decreased expression in the wildtype (Fig. 5b).

To gain insight into the biology affected by the expression changes observed in the *Pten*^*Y68H/*+^ cortex, we performed Ingenuity Pathway Analysis (IPA), which identified the top “canonical pathways” that show enrichment beyond random chance based on the input gene list, i.e., the genes showing differential expression (threshold: *P* < 0.05; Log_2_(Fold Change) ≥ 1.0 or Log_2_(Fold Change) ≤ -1.0). The top ten pathways are all related to cellular stress and inflammation signaling (Fig. 5c). This signature is driven by the differential expression of *Card10*, *Il1r1*, *Ngfr*, *Tcf7l2*, and *Ttr*, where *Il1r1*, the interleukin 1 receptor type 1 gene, appears in the associated lists of 90% of the pathways (Fig. 5c). The top network showing how the differentially expressed molecules are biologically related implicates *Tcf7l2* as an important regulatory node given that it has the highest degree centrality (i.e., 12) in the network (Fig. 5d). Furthermore, using STRING analysis, an important gene–gene association network was identified from among the differentially expressed genes, implicating oxytocinergic signaling. Differential expression analysis found a roughly fivefold increase in oxytocin (*Oxt*), and network analysis of associated genes showing DE found a small network where *Oxt* has the highest degree and betweenness centrality (Fig. 5e; Additional file 2).

To expand our transcriptomic analysis of the *Pten*^*Y68H/*+^ cortex, we utilized a NanoString nCounter® Glial Profiling Panel comparing *Pten*^*Y68H/*+^ and *Pten*^+*/*+^ mice (*N* = 8). We found that the expression of glial-related genes as assessed by the NanoString panel was consistent with our RNA sequencing experiments and our cellular phenotyping on microglial. We again found that *C1q*, *Iba1*, *Cx3cr1*, and *Csf1r* were upregulated in the *Pten*^*Y68H/*+^ cortex (Additional file 1: Fig. S5a). In subjecting the differentially expressed genes to IPA pathway analysis, we found the enriched pathways associated with neuroinflammation, autophagy, phagosome maturation, and complement signaling significantly increased in expression in *Pten*^*Y68H/*+^ (Additional file 1: Fig. S5b). Subsequent network analysis also implicated other genes known to be dysregulated, *Dap12* and *Trem2* were also implicated in the network of complement and neuroinflammatory genes dysregulated in the *Pten*^*Y68H/*+^ (Additional file 1: Fig. S5c). This more specified analysis of gene expression provided supporting validation of our observation of microglial activation and the generalized neuroinflammatory signature in the neural transcriptome.

### Increased Oxytocin expression in paraventricular neurons in ***Pten***^***Y68H/***+^ hypothalamus

**Fig. 6:**
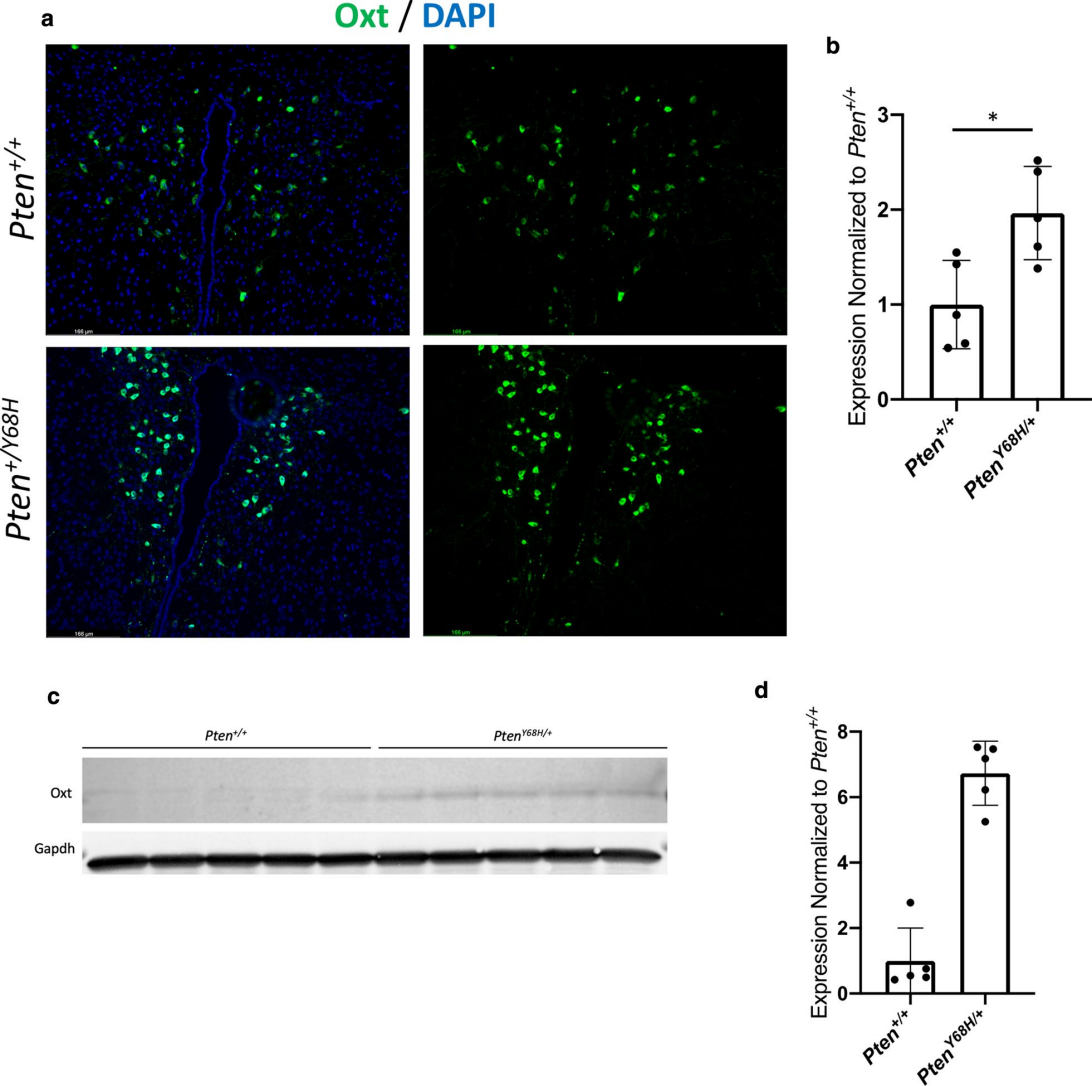
Overexpression of oxytocin in paraventricular nucleus (PVN) of the hypothalamus of six-week-old *Pten*^+*/*+^ and *Pten*^*Y68H/*+^ mice. **a** Representative immunofluorescence staining of six-week-old *Pten*^+*/*+^ and *Pten*^*WT/Y68H*^ PVN for Oxt (green) and DAPI (blue). *N* = 3. Magnification = 10X. Scale bar = 166 μm. **b** Quantification of the integrated density of Oxt stain per biological replicate, finding a significant increase in Oxt in the *Pten*^*Y68H/*+^ PVN compared to *Pten*^+*/*+^ PVN (*P* = 0.032). **c** Western analysis for Oxt expression in of six-week-old *Pten*^+*/*+^ and *Pten*^*Y68H/*+^ hypothalamus (*N* = 5). **d** Quantification of Western in panel c (*P* = 0.0079). *P* value key: *P < 0.05, **P < 0.01

Given the importance of oxytocin signaling to social behavior, we sought to understand more about the possible biological effects of increased expression of *Oxt*. Thus, we deployed IPA’s Molecule Activity Predictor (MAP) to understand how the fivefold increase in *Oxt* may affect downstream interactors. From the top 10 molecules directly downstream of *Oxt*, we found that increased *Oxt* expression predicts an increase in dopamine, calcium, Prkcz, and Egfr activity and a decrease in Crh and Fos activity (Fig. 5f). Finally, we sought to confirm the increase in *Oxt* expression in the brain of *Pten*^*Y68H/*+^ mice so we performed immunofluorescence staining for Oxt in P40 hypothalamus. By visual inspection alone, we found a dramatic increase in Oxt expression in the paraventricular neurons (PVN) of the hypothalamus of P40 *Pten*^*Y68H/*+^ mice (*N* = 5; Fig. 6a). In addition, we plotted the average global expression of Oxt per biological replicate to show these data were not skewed (Median_Δ_ = 0.98; 97% CI: 0.063–1.86; *P* = 0.032; Fig. 6b). To validate the increased hypothalamic Oxt expression, we performed Western analysis. This confirmed Oxt overexpression in the PVN of *Pten*^*Y68H*^ mice (Median_Δ_ = 5.8; 97% CI: 4.4–7.0; *P* = 0.0079; Fig. 6c, d). Given these data, nuclear-predominant Pten, *Pten*^*Y68H*^*,* associates with increased Oxt expression in the brain.

## Discussion

In this study, we demonstrate that the nuclear-predominant *Pten*^*Y68H*^ mutation in mice results in decreased sociability, decreased interest in novel social stimuli, increased perseverative behavior, likely increased anxiety, increased microglial activation, increased expression of neuroinflammatory genes, and increased neural oxytocin levels. We show that in addition to deficits in social behavior (Fig. 2), *Pten*^*Y68H/*+^ mice have macrocephaly from increased brain mass, and this is associated with nuclear enrichment of Pten, decreased Pten expression, and increased phosphorylation of Akt (Fig. 1). Moreover, we show that *Pten*^*Y68H/*+^ microglia are activated in vivo*,* expressing elevated amounts microglial, inflammatory, and neurotrophic markers, including C1q (Fig. 3). We investigated the cellular origins of the microglial pathology finding that it appears to arise cell autonomously and also enhances phagocytic activity (Fig. 4). To understand the molecular landscape undergirding these cellular and behavioral pathologies, we performed a transcriptomic survey of the cortex of *Pten*^*Y68H/*+^ mice identifying broad changes in gene expression much of which implicates neuroinflammatory or other neurological pathways, including the striking finding of increased oxytocin expression (Fig. 5). The oxytocin expression finding was confirmed at the protein level via staining and Western analysis of the hypothalamus (Fig. 6). Together these data implicate Pten localization and expression as an important regulator of behavior, microglia activation and phagocytosis, neuroinflammatory genes expression and the oxytocin system.

Murine models of increasing specificity have established that Pten and related downstream signaling participate in the regulation of social behavior, CNS morphology, and neuronal and glial function [22, 24, 44–65]. Consistent behavioral phenotypes of altered social behavior persist when loss of Pten expression is restricted to mature neurons or neuronal precursors, and these models often have impaired learning/memory, increased anxiety, and/or altered activity/motor ability [15, 44, 46, 48]. These behavioral abnormalities contrast somewhat with those observed in our germline *Pten*^*m3m4*^, cytoplasmic-predominant Pten mislocalization model [22]. *Pten*^*m3m4/m3m4*^ mice maintain relatively normal capacities for learning and memory, while showing a sex-specific (i.e., male) increase in sociability but severely impaired motor coordination; motor coordination deficits occur in male and female *Pten*^*m3m4/m3m4*^ mice [15, 22]. *Pten*^*Y68H/*+^ mice show decreased sociability and interest in social novelty with increased repetitive behavior and possible anxiety. It is difficult to assert confidently the sources of variability in the behavioral phenotypes observed in these various *Pten* mouse models; however, the *Pten*^*m3m4/m3m4*^ and *Pten*^*Y68H/*+^ models are distinct from *Pten* knock-out or conditional knock-out models in that the mutations are designed to disrupt Pten localization instead of completely eliminating all Pten expression and thus functionality. Of course, these same missense models are still complicated by the frequent protein instability and increased proteasomal degradation associated with many missense mutations [43].

The *Pten*^*m3m4*^ and *Pten*^*Y68H*^ mutations simulate or copy mutations observed in PTEN-ASD individuals, respectively. In terms of the behavioral differences between the *Pten*^*m3m4/m3m4*^ and *Pten*^*Y68H/*+^ models, it is also difficult to confidently attribute certain behaviors to Pten localization partially because both mutations disrupt Pten stability and phosphatase activity to some extent; for instance, we know that the *Pten*^*Y68H*^ mutation has a very damaging effect on the stability of Pten [43]. In fact, we have shown decreased Pten expression over time in both our models [22, 30]. However, it is worth noting that the cytoplasmic versus nuclear localization models appear to associate with contrasting effects on sociability, indicating a clear possibility that subcellular localization effects may be important. It is likely that additional models and elegantly designed experiments will be needed to accurately and robustly associate Pten localization changes with specific behavioral changes in mice.

Beyond the behavioral phenotypes, our contrasting *Pten* models do reveal something about the effects of Pten localization on glial phenotypes. The *Pten*^*m3m4/m3m4*^ mouse has aggressive oligodendrocyte and astrocyte pathologies, including increased myelination [22, 29], whereas the *Pten*^*Y68H/*+^ mouse has no apparent oligodendrocyte or astrocyte pathologies (Additional file 1: Fig S2A-H). However, both the *Pten*^*m3m4/m3m4*^ and *Pten*^*Y68H/*+^ mice have activated microglia with enhanced phagocytic activity. Comparatively, these findings suggest that the localization affects glial phenotypes, where less nuclear Pten provokes oligodendrocyte and astrocyte pathologies. On the other hand, localization appears to not have an effect on the behavior of microglia; rather, the total steady-state level of Pten appears to regulate microglia function. Both the *Pten*^*m3m4*^ and *Pten*^*Y68H*^ mutations disrupt Pten stability and both have activated microglia, a pathology shown to be of cell autonomous origins. Glia development and function appear to be tied closely to various aspects of Pten activity, presenting a likely fruitful area of future study.

Possibly the most surprising and important finding of this study is the observation of increased oxytocin expression in the *Pten*^*Y68H/*+^ brain, which occurs in a model with clear behavioral abnormalities. A wealth of research, including extensive animal modeling, demonstrates persuasively that the oxytocin system is important to prosocial cognition [62]. In fact, research has shown that oxytocin may be a viable therapeutic modality for ameliorating social deficits in individuals with ASD [62–65]. Interestingly, many of the models referenced above (e.g., *Oxt* knockouts) have deficits in the oxytocin system where mice either have lower circulating Oxt or an inability to respond to exogenous Oxt due to knockout (i.e., oxytocin receptor knockout). Our molecular phenotype for oxytocin is quite the opposite. This may seem paradoxical that a mouse with behavioral deficits has an elevated amount of a prosocial neuropeptide. However, it has been shown that high exogenous doses of oxytocin can paradoxically provoke an anxiogenic response due to the excess oxytocin, after oxytocin receptor (OXTR) saturation, acting on vasopressin receptors [64–67]. Moreover, it has also been observed in other models, such as the BTBR mouse, that increased oxytocin expression and social deficits (i.e., increased anxiety) can co-occur [68]. Alternatively, it is possible that *Pten*^*Y68H*^ mice are somehow insensitive to Oxt (despite normal Oxtr expression) and thus the increased Oxt is a compensatory response. Regardless of the molecular etiology, we believe that the increased *Oxt* expression observed in the RNA-sequencing experiment (Fig. 5) and confirmed in the PVN (Fig. 6) is likely relevant to some of the behavioral abnormalities observed in the *Pten*^*Y68H/*+^ mouse. This is important as this is the first time the oxytocin system has be observed as perturbed in a *Pten* model. Disruption of the oxytocin system may be specific to only a subset of *Pten* mutations, such as those that cause nuclear mislocalization. More research is required to sort out whether oxytocin system problems are common to all *Pten* models or only to a specific subset of mutations.

## Limitations

The strengths of our study are founded on the specificity of the mouse modeling (i.e., a knockin mutation identical to that observed in PTEN-ASD individuals) and the rigorous behavioral, cellular, and molecular phenotyping. However, there are limitations to our *Pten*^*Y68H/*+^ model, such as the deleterious effect of the Y68H mutation on the stability and overall steady-state level of Pten [33, 42]. The decreased stability of *Pten*^*Y68H*^ makes it difficult to absolutely attribute causality of any phenotype to the localization changes observed. Moreover, the localization changes themselves are not absolute either. As clearly shown in Fig. 1b, c, the change in localization is relative, and there is still plenty Pten in the cytoplasm; However, the extent to which the cytoplasmic Pten is of wildtype or mutant providence is unknown as the germline nature of the model since the *Pten*^*Y68H/Y68H*^ genotype exhibits embryonic lethality, therefore requiring the study of only heterozygous mutants. It is unclear whether the Y68H mutation can function as a dominant-negative mutant as has been described for other *PTEN* missense mutations [69], but there is some existing evidence to suggest that it does not function as such [27, 42, 70]. Future work designed to specifically interrogate questions about the functional effects of Pten localization on social and neurobiological phenotypes will have to utilize more extensive modeling strategies. Additionally, the phenotyping of this model, especially behaviorally, can always be expanded upon for a deeper and more nuanced understanding of the behavior deficits; however, our work at least makes clear significant deficits exist. However, the *Pten*^*Y68H/*+^ model is extremely useful for gaining important insights into these challenging and important scientific questions about pathophysiology, which should help inform monogenic ASD risk cases.

## Conclusion

Although there, of course, remains much to explore about the *Pten*^*Y68H/*+^ mouse and other *Pten* models, especially in terms of the effects on social cognition and neurobiology, this study is an important step toward understanding how Pten localization in the brain can affect social cognition and neuronal and glial function. Until this study, it was unclear whether decreased cytoplasmic expression of Pten would affect the CNS in such a dramatic fashion. Our work on the *Pten*^*Y68H/*+^ mouse demonstrates that social behavior can be modulated by mutations that shift Pten to the cytoplasm or nucleus, but the exact social phenotypes can be quite distinct. Contrastingly, certain glial phenotypes seem to be in part dependent on Pten localization as the *Pten*^*Y68H/*+^ mouse shows no apparent changes in oligodendrocytes and astrocytes (Additional file 1: Fig S2), whereas its complement, the *Pten*^*m3m4*^ model, show aggressive glial pathologies [22, 29]. However, microglial dysfunction seems to be entirely independent of Pten localization, instead resulting from decreased Pten expression levels in general. In addition, we find prominent oxytocin overexpression in the hypothalamus of *Pten*^*Y68H/*+^ mice, thus linking *Pten* mutation and the oxytocin system for the first time. In sum, this study demonstrates the importance of nuclear Pten to CNS morphology and function, while linking Pten, a prominent ASD risk gene, to a neuroendocrine modulator of social behavior, oxytocin, in a murine model with clear social deficits.

## Supplementary Information

**Additional file 1.** contains QC information on the transcriptomic study, glia phenotyping data, NanoString experimental data, and qRT PCR validation of selected RNA-Seq hits.

**Additional file 2.** contains the raw differential expression data from transcriptomic surgery of P40 Y68H cortex.

## Data Availability

The datasets for this study are available from the corresponding author on reasonable request.

## Abbreviations

ASD: Autism spectrum disorder
CNS: Central nervous system
OXT: Oxytocin
PTEN: Phosphatase and Tensin Homolog
PHTS: PTEN Hamartoma Tumor Syndrome

## References

[1] Butler M, Dasouki M, Zhou X, Talebizadeh Z, Brown M, Takahashi T (2005). Subset of individuals with autism spectrum disorders and extreme macrocephaly associated with germline PTEN tumour suppressor gene mutations. J Med Genet.

[2] Buxbaum JD, Cai G, Chaste P, Nygren G, Goldsmith J, Reichert J (2007). Mutation screening of the PTEN gene in patients with autism spectrum disorders and macrocephaly. Am J Med Genet Part B Neuropsychiatr Genet Off Publ Int Soc Psychiatr Genet.

[3] Varga EA, Pastore M, Prior T, Herman GE, McBride KL (2009). The prevalence of PTEN mutations in a clinical pediatric cohort with autism spectrum disorders, developmental delay, and macrocephaly. Genet Med Off J Am Coll Med Genet.

[4] Orrico A, Galli L, Buoni S, Orsi A, Vonella G, Sorrentino V (2009). Novel PTEN mutations in neurodevelopmental disorders and macrocephaly. Clin Genet.

[5] McBride KL, Varga EA, Pastore MT, Prior TW, Manickam K, Atkin JF (2010). Confirmation study of PTEN mutations among individuals with autism or developmental delays/mental retardation and macrocephaly. Autism Res Off J Int Soc Autism Res.

[6] O’Roak BJ, Deriziotis P, Lee C, Vives L, Schwartz JJ, Girirajan S (2011). Exome sequencing in sporadic autism spectrum disorders identifies severe de novo mutations. Nat Genet.

[7] O’Roak BJ, Vives L, Fu W, Egertson JD, Stanaway IB, Phelps IG (2012). Multiplex targeted sequencing identifies recurrently mutated genes in autism spectrum disorders. Science.

[8] O’Roak BJ, Vives L, Girirajan S, Karakoc E, Krumm N, Coe BP (2012). Sporadic autism exomes reveal a highly interconnected protein network of de novo mutations. Nature.

[9] Klein S, Sharifi-Hannauer P, Martinez-Agosto JA (2013). Macrocephaly as a clinical indicator of genetic subtypes in autism. Autism Res Off J Int Soc Autism Res.

[10] De Rubeis S, He X, Goldberg AP, Poultney CS, Samocha K, Cicek AE (2014). Synaptic, transcriptional and chromatin genes disrupted in autism. Nature.

[11] Hobert JA, Embacher R, Mester JL, Frazier TW, Eng C (2014). Biochemical screening and PTEN mutation analysis in individuals with autism spectrum disorders and macrocephaly. Eur J Hum Genet EJHG.

[12] Marchese M, Conti V, Valvo G, Moro F, Muratori F, Tancredi R (2014). Autism-epilepsy phenotype with macrocephaly suggests PTEN, but not GLIALCAM, genetic screening. BMC Med Genet.

[13] Satterstrom FK, Kosmicki JA, Wang J, Breen MS, De Rubeis S, An J-Y (2020). Large-scale exome sequencing study implicates both developmental and functional changes in the neurobiology of autism. Cell.

[14] Fombonne E, Rogé B, Claverie J, Courty S, Frémolle J (1999). Microcephaly and macrocephaly in autism. J Autism Dev Disord.

[15] Tilot AK, Frazier TW, Eng C (2015). Balancing proliferation and connectivity in pten-associated autism spectrum disorder. Neurother J Am Soc Exp Neurother.

[16] Yehia L, Ngeow J, Eng C (2019). PTEN-opathies: from biological insights to evidence-based precision medicine. J Clin Invest.

[17] Marsh DJ, Kum JB, Lunetta KL, Bennett MJ, Gorlin RJ, Ahmed SF (1999). PTEN mutation spectrum and genotype-phenotype correlations in Bannayan–Riley–Ruvalcaba syndrome suggest a single entity with Cowden syndrome. Hum Mol Genet.

[18] Maehama T, Dixon JE (1998). The tumor suppressor, PTEN/MMAC1, dephosphorylates the lipid second messenger, phosphatidylinositol 3,4,5-trisphosphate. J Biol Chem.

[19] Worby CA, Dixon JE (2014). PTEN. Annu Rev Biochem.

[20] Hasle N, Matreyek KA, Fowler DM (2019). The impact of genetic variants on PTEN molecular functions and cellular phenotypes. Cold Spring Harb Perspect Med.

[21] Ho J, Cruise ES, Dowling RJO, Stambolic V (2020). PTEN nuclear functions. Cold Spring Harb Perspect Med.

[22] Tilot AK, Gaugler MK, Yu Q, Romigh T, Yu W, Miller RH (2014). Germline disruption of Pten localization causes enhanced sex-dependent social motivation and increased glial production. Hum Mol Genet.

[23] Tilot AK, Bebek G, Niazi F, Altemus J, Romigh T, Frazier TW (2016). Neural transcriptome of constitutional Pten dysfunction in mice and its relevance to human idiopathic Autism Spectrum Disorder. Mol Psychiatry.

[24] Fricano-Kugler CJ, Getz SA, Williams MR, Zurawel AA, DeSpenza T, Frazel PW (2018). Nuclear excluded autism-associated phosphatase and tensin homolog mutations dysregulate neuronal growth. Biol Psychiatry.

[25] Frazier TW, Embacher R, Tilot AK, Koenig K, Mester J, Eng C (2015). Molecular and phenotypic abnormalities in individuals with germline heterozygous PTEN mutations and autism. Mol Psychiatry.

[26] Leslie NR, Longy M (2016). Inherited PTEN mutations and the prediction of phenotype. Semin Cell Dev Biol.

[27] Mighell TL, Thacker S, Fombonne E, Eng C, O’Roak BJ (2020). An Integrated deep-mutational-scanning approach provides clinical insights on PTEN genotype-phenotype relationships. Am J Hum Genet.

[28] Yehia L, Keel E, Eng C (2020). The clinical spectrum of PTEN mutations. Annu Rev Med.

[29] Lee H, Thacker S, Sarn N, Dutta R, Eng C (2019). Constitutional mislocalization of Pten drives precocious maturation in oligodendrocytes and aberrant myelination in model of autism spectrum disorder. Transl Psychiatry.

[30] Sarn N, Jaini R, Thacker S, Lee H, Dutta R, Eng C. Cytoplasmic-predominant Pten increases microglial activation and synaptic pruning in a murine model with autism-like phenotype. Mol Psychiatry. 2020; 10.1038/s41380-020-0681-0 PMC8159731 32055008

[31] Kang SC, Jaini R, Hitomi M, Lee H, Sarn N, Thacker S (2020). Decreased nuclear Pten in neural stem cells contributes to deficits in neuronal maturation. Mol Autism.

[32] Lobo GP, Waite KA, Planchon SM, Romigh T, Nassif NT, Eng C (2009). Germline and somatic cancer-associated mutations in the ATP-binding motifs of PTEN influence its subcellular localization and tumor suppressive function. Hum Mol Genet.

[33] He X, Ni Y, Wang Y, Romigh T, Eng C (2011). Naturally occurring germline and tumor-associated mutations within the ATP-binding motifs of PTEN lead to oxidative damage of DNA associated with decreased nuclear p53. Hum Mol Genet.

[34] Yang M, Silverman JL, Crawley JN. Automated three-chambered social approach task for mice. Curr Protoc Neurosci. 2011;Chapter 8:Unit 8.26. 10.1002/0471142301.ns0826s56 PMC4904775 21732314

[35] Angoa-Pérez M, Kane MJ, Briggs DI, Francescutti DM, Kuhn DM (2013). Marble burying and nestlet shredding as tests of repetitive, compulsive-like behaviors in mice. J Vis Exp JoVE.

[36] Dobin A, Gingeras TR (2015). Mapping RNA-seq Reads with STAR. Curr Protoc Bioinforma.

[37] Dobin A, Gingeras TR (2016). Optimizing RNA-Seq mapping with STAR. Methods Mol Biol Clifton NJ.

[38] Dobin A, Davis CA, Schlesinger F, Drenkow J, Zaleski C, Jha S (2013). STAR: ultrafast universal RNA-seq aligner. Bioinforma Oxf Engl.

[39] Geiss GK, Bumgarner RE, Birditt B, Dahl T, Dowidar N, Dunaway DL (2008). Direct multiplexed measurement of gene expression with color-coded probe pairs. Nat Biotechnol.

[40] Mester JL, Tilot AK, Rybicki LA, Frazier TW, Eng C (2011). Analysis of prevalence and degree of macrocephaly in patients with germline PTEN mutations and of brain weight in Pten knock-in murine model. Eur J Hum Genet EJHG.

[41] Tan M-H, Mester J, Peterson C, Yang Y, Chen J-L, Rybicki LA (2011). A clinical scoring system for selection of patients for PTEN mutation testing is proposed on the basis of a prospective study of 3042 probands. Am J Hum Genet.

[42] Association AP. Diagnostic and statistical manual of mental disorders (DSM-5®). American Psychiatric Pub; 2013. 10.1590/s2317-17822013000200017 24413388

[43] Matreyek KA, Starita LM, Stephany JJ, Martin B, Chiasson MA, Gray VE (2018). Multiplex assessment of protein variant abundance by massively parallel sequencing. Nat Genet.

[44] Kwon C-H, Zhou J, Li Y, Kim KW, Hensley LL, Baker SJ (2006). Neuron-specific enolase-cre mouse line with cre activity in specific neuronal populations. Genes.

[45] Ogawa S, Kwon C-H, Zhou J, Koovakkattu D, Parada LF, Sinton CM (2007). A seizure-prone phenotype is associated with altered free-running rhythm in Pten mutant mice. Brain Res.

[46] Zhou J, Blundell J, Ogawa S, Kwon C-H, Zhang W, Sinton C (2009). Pharmacological inhibition of mTORC1 suppresses anatomical, cellular, and behavioral abnormalities in neural-specific Pten knock-out mice. J Neurosci Off J Soc Neurosci.

[47] Page DT, Kuti OJ, Prestia C, Sur M (2009). Haploinsufficiency for Pten and Serotonin transporter cooperatively influences brain size and social behavior. Proc Natl Acad Sci USA.

[48] Amiri A, Cho W, Zhou J, Birnbaum SG, Sinton CM, McKay RM (2012). Pten deletion in adult hippocampal neural stem/progenitor cells causes cellular abnormalities and alters neurogenesis. J Neurosci Off J Soc Neurosci.

[49] Kazdoba TM, Sunnen CN, Crowell B, Lee GH, Anderson AE, D’Arcangelo G (2012). Development and characterization of NEX- Pten, a novel forebrain excitatory neuron-specific knockout mouse. Dev Neurosci.

[50] Clipperton-Allen AE, Page DT (2014). Pten haploinsufficient mice show broad brain overgrowth but selective impairments in autism-relevant behavioral tests. Hum Mol Genet.

[51] Clipperton-Allen AE, Chen Y, Page DT (2016). Autism-relevant behaviors are minimally impacted by conditional deletion of Pten in oxytocinergic neurons. Autism Res Off J Int Soc Autism Res.

[52] Lugo JN, Smith GD, Morrison JB, White J (2013). Deletion of PTEN produces deficits in conditioned fear and increases fragile X mental retardation protein. Learn Mem Cold Spring Harb N.

[53] Lugo JN, Smith GD, Arbuckle EP, White J, Holley AJ, Floruta CM (2014). Deletion of PTEN produces autism-like behavioral deficits and alterations in synaptic proteins. Front Mol Neurosci.

[54] Chen Y, Huang W-C, Séjourné J, Clipperton-Allen AE, Page DT (2015). Pten mutations alter brain growth trajectory and allocation of cell types through elevated β-catenin signaling. J Neurosci Off J Soc Neurosci.

[55] Clipperton-Allen AE, Page DT (2015). Decreased aggression and increased repetitive behavior in Pten haploinsufficient mice. Genes Brain Behav.

[56] Igarashi A, Itoh K, Yamada T, Adachi Y, Kato T, Murata D (2018). Nuclear PTEN deficiency causes microcephaly with decreased neuronal soma size and increased seizure susceptibility. J Biol Chem.

[57] Chen C-J, Sgritta M, Mays J, Zhou H, Lucero R, Park J (2019). Therapeutic inhibition of mTORC2 rescues the behavioral and neurophysiological abnormalities associated with Pten-deficiency. Nat Med.

[58] Clipperton-Allen AE, Cohen OS, Aceti M, Zucca A, Levy J, Ellegood J, et al. *Pten* haploinsufficiency disrupts scaling across brain areas during development in mice. Transl Psychiatry. http://feeds.nature.com/~r/tp/rss/current/~3/TaNbTgrnJSQ/s41398-019-0656-6?utm_source=researcher_app&utm_medium=referral&utm_campaign=RESR_MRKT_Researcher_inbound 10.1038/s41398-019-0656-6 PMC6895202 31804455

[59] Skelton PD, Frazel PW, Lee D, Suh H, Luikart BW. Pten loss results in inappropriate excitatory connectivity. Mol Psychiatry. 2019 Apr 9 [cited 2019 Apr 15]; http://www.nature.com/articles/s41380-019-0412-6 10.1038/s41380-019-0412-6 PMC6785382 30967683

[60] Skelton PD, Stan RV, Luikart BW (2020). The Role of PTEN in Neurodevelopment. Mol Neuropsychiatry.

[61] Skelton PD, Poquerusse J, Salinaro JR, Li M, Luikart BW (2020). Activity-dependent dendritic elaboration requires Pten. Neurobiol Dis.

[62] Modi ME, Young LJ (2012). The oxytocin system in drug discovery for autism: animal models and novel therapeutic strategies. Horm Behav.

[63] Andari E, Duhamel J-R, Zalla T, Herbrecht E, Leboyer M, Sirigu A (2010). Promoting social behavior with oxytocin in high-functioning autism spectrum disorders. Proc Natl Acad Sci.

[64] Preckel K, Kanske P, Singer T, Paulus FM, Krach S (2016). Clinical trial of modulatory effects of oxytocin treatment on higher-order social cognition in autism spectrum disorder: a randomized, placebo-controlled, double-blind and crossover trial. BMC Psychiatry.

[65] Quintana DS, Westlye LT, Hope S, Nærland T, Elvsåshagen T, Dørum E (2017). Dose-dependent social-cognitive effects of intranasal oxytocin delivered with novel Breath Powered device in adults with autism spectrum disorder: a randomized placebo-controlled double-blind crossover trial. Transl Psychiatry.

[66] Huang H, Michetti C, Busnelli M, Managò F, Sannino S, Scheggia D (2014). Chronic and acute intranasal oxytocin produce divergent social effects in mice. Neuropsychopharmacology.

[67] Preckel K, Kanske P (2018). Amygdala and oxytocin functioning as keys to understanding and treating autism: Commentary on an RDoC based approach. Neurosci Biobehav Rev.

[68] Silverman JL, Yang M, Turner SM, Katz AM, Bell DB, Koenig JI (2010). Low stress reactivity and neuroendocrine factors in the BTBR T+tf/J mouse model of autism. Neuroscience.

[69] Papa A, Wan L, Bonora M, Salmena L, Song MS, Hobbs RM (2014). Cancer-associated PTEN mutants act in a dominant negative manner to suppress PTEN protein function. Cell.

[70] Mighell TL, Evans-Dutson S, O’Roak BJ (2018). A saturation Mutagenesis approach to understanding PTEN lipid phosphatase activity and genotype-phenotype relationships. Am J Hum Genet.

