# Supplementary material for: Germline nuclear-predominant Pten murine model exhibits impaired social and perseverative behavior, microglial activation, and increased oxytocinergic activity
Source: Mol Autism. 2021 Jun 4;12:41. doi: 10.1186/s13229-021-00448-4 (PMC8176582; doi:10.1186/s13229-021-00448-4)
Supplement: Supplementary file 1 — Additional file 1. contains QC information on the transcriptomic study, glia phenotyping data, NanoString experimental data, and qRT PCR validation of selected RNA-Seq hits. [file 13229_2021_448_MOESM1_ESM.docx]

**Additional File 1: Supplementary Information**

**Germline, Nuclear-Predominant Pten Murine Model Exhibits Impaired Social and Perseverative Behavior, Microglial Activation, and Increased Oxytocinergic Activity**

Nick Sarn^1,2,¥^, Stetson Thacker^1,3,¥^, Hyunpil Lee^1^, Charis Eng^1-5*^

^1^Genomic Medicine Institute, Lerner Research Institute, Cleveland Clinic, Cleveland, OH, 44195, USA

^2^Department of Genetics and Genome Sciences, Case Western Reserve University School of Medicine, Cleveland, OH, 44106, USA

^3^Cleveland Clinic Lerner College of Medicine, Case Western Reserve University, Cleveland, OH, 44195, USA

^4^Case Comprehensive Cancer Center, Case Western Reserve University School of Medicine, Cleveland, OH, 44106, USA

^5^Taussig Cancer Institute, Cleveland Clinic, Cleveland, OH, 44195, USA

^¥^These authors contributed equally

*Correspondence: [engc@ccf.org](mailto:engc@ccf.org); Genomic Medicine Institute, Lerner Research Institute, Cleveland Clinic 9500 Euclid Avenue, Cleveland, OH 44195; Tel: (216) 444-3900

Authors’ contact information: Nick Sarn ([sarnn@ccf.org](mailto:sarnn@ccf.org)); Stetson Thacker ([thackes@ccf.org](mailto:thackes@ccf.org)); Hyunpil Lee ([leeh6@ccf.org](mailto:leeh6@ccf.org)); Charis Eng ([engc@ccf.org](mailto:engc@ccf.org))


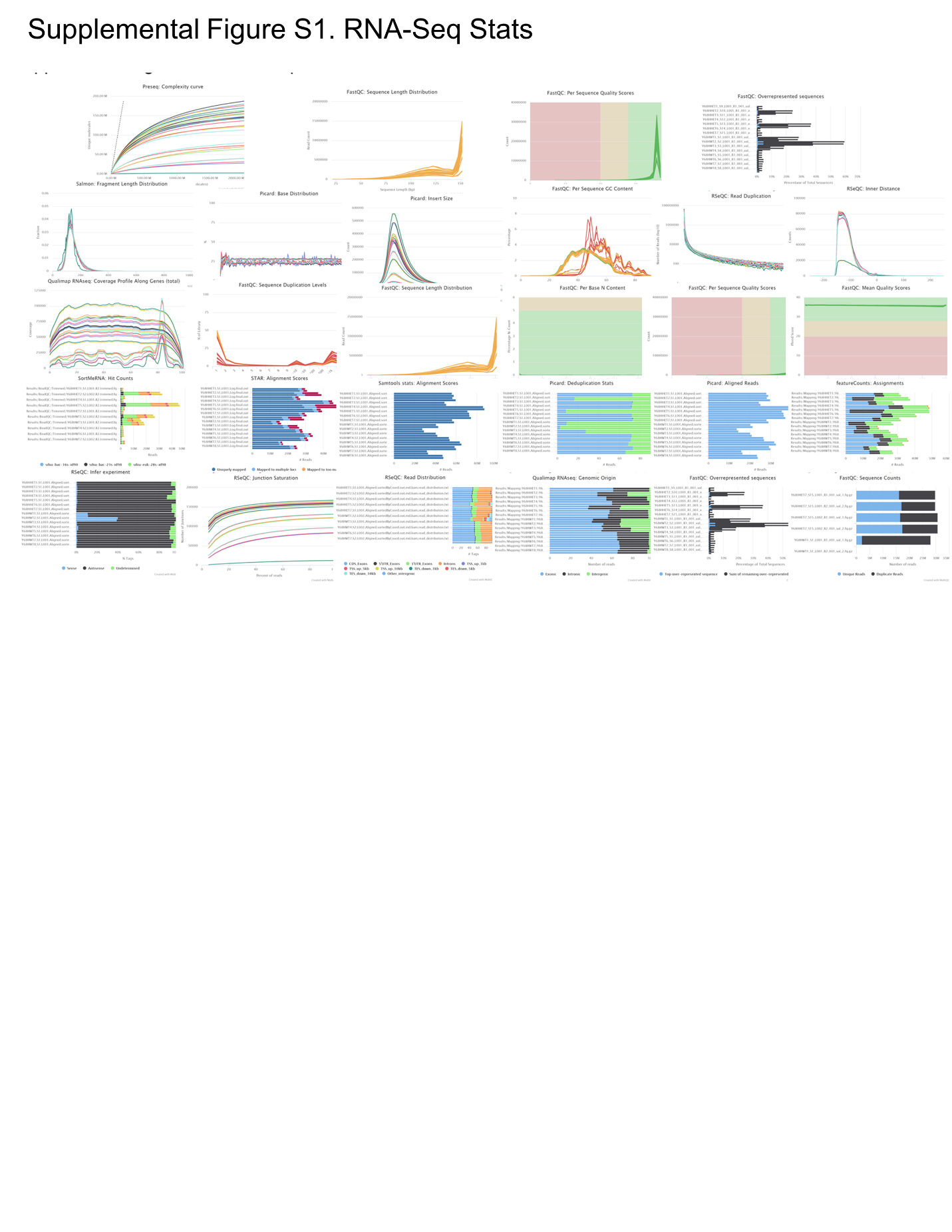


**Supplemental Figure S1.** Quality control output from MultiQC analysis of *Pten^+/+^* and *Pten^Y68H/+^* trimmed fastq files, bam files subsequent to STAR alignment, and fastq files indexed with Salmon. N = 15.


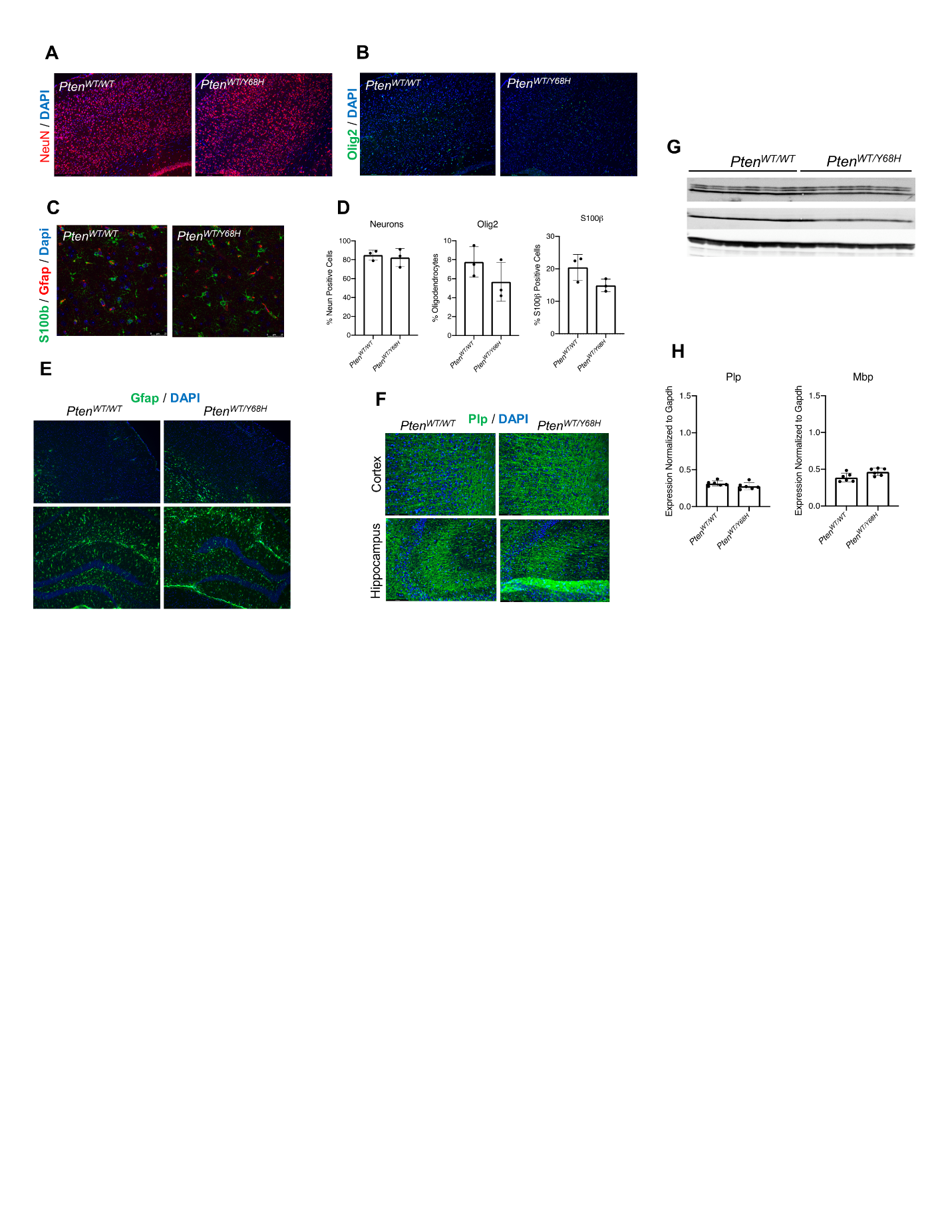


**Supplemental Figure S2.** Cellular phenotyping of *Pten^Y68H/+^* neurons and glia **a** Immunofluorescence staining of P180 (i.e. six-months-old) cortical sections staining neurons with NeuN (red) and DAPI (blue). **b** Immunofluorescence staining of P180 cortical sections staining oligodendrocytes with Olig1 (green) with DAPI (blue). **c** Immunofluorescence staining of P180 cortical slides for astrocytes with s100β (green), Gfap (red) with DAPI (blue)**.** **d** Quantification of staining (% cell-specific positive marker/total DAPI positive cells) from panels a-c. **d** Immunofluorescence staining of P180 brain sections staining astrocytes for Gfap (green) with DAPI (blue) in cortex (top panels) and hippocampus (bottom panels)**.**  **f** and **e** Immunofluorescence staining of P180 brain sections staining myelin tracks for Plp (green) with DAPI (blue) in cortex (top panels) and Hippocampus (bottom panels)**. g** Western blot analysis of P180 cortical lysates from *Pten^+/+^* (*Pten^WT/WT^*) and *Pten^Y68H/+^* (*Pten^WT/Y68H^*) mice on myelin markers Plp and Mbp. **H** Densitometric quantification of Plp and Mbp normalized to Gapdh Panel g.


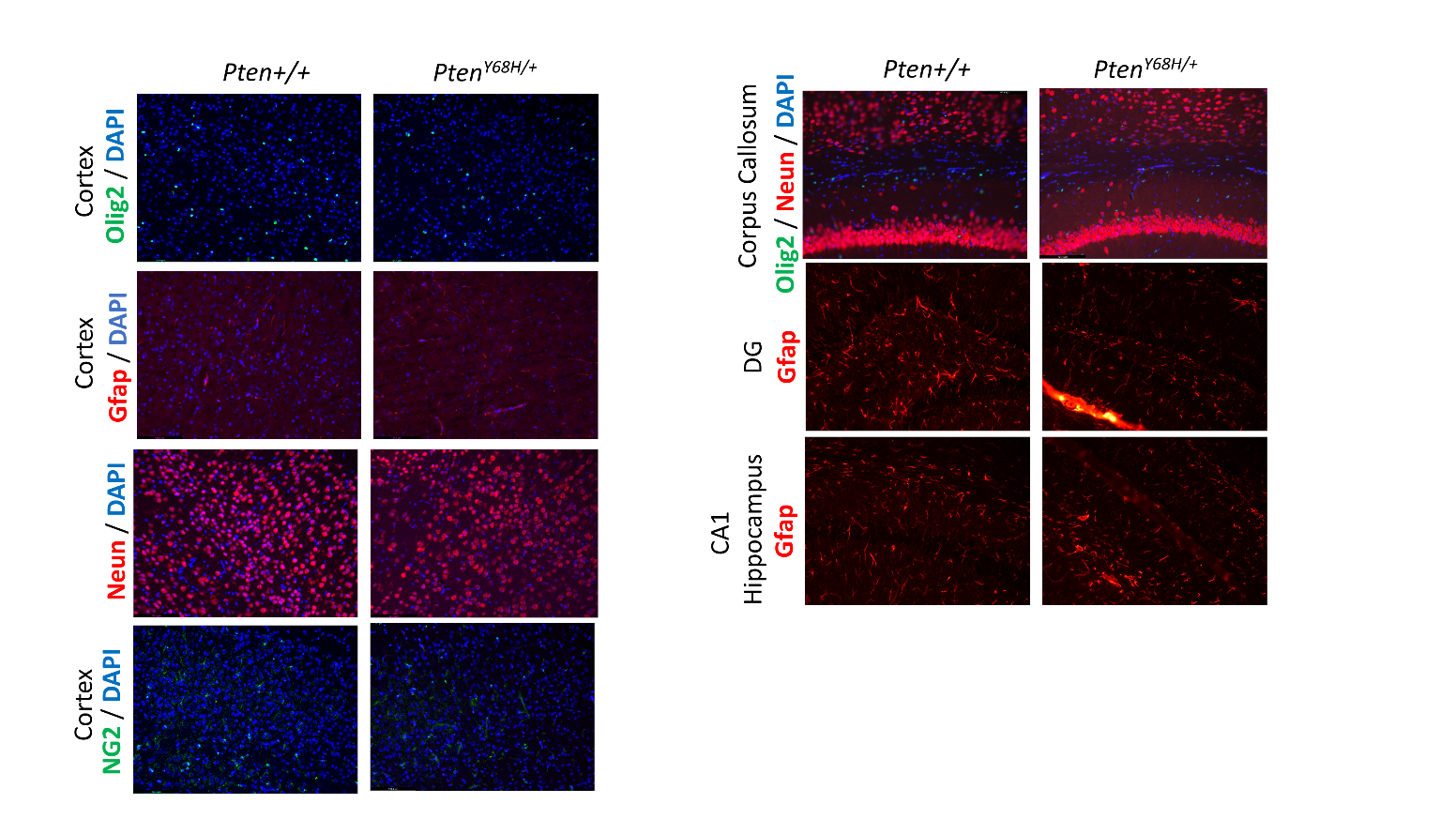


**Supplemental Figure S3.** Cellular phenotyping of *Pten^Y68H/+^* neurons and glia at P40. **a** IF on cortical sections from P40 mice, assessing oligodendrocyte (Olig1), astrocyte (Gfap), neurons (NeuN), and oligodendrocyte progenitor cells (Ng2). No differences observed in these glia. Consistent with P180 observations. **b** IF on sections from P40 corpus callosum, CA1 hippocampus, and dentate gyrus assessing various glial markers. No differences observed.


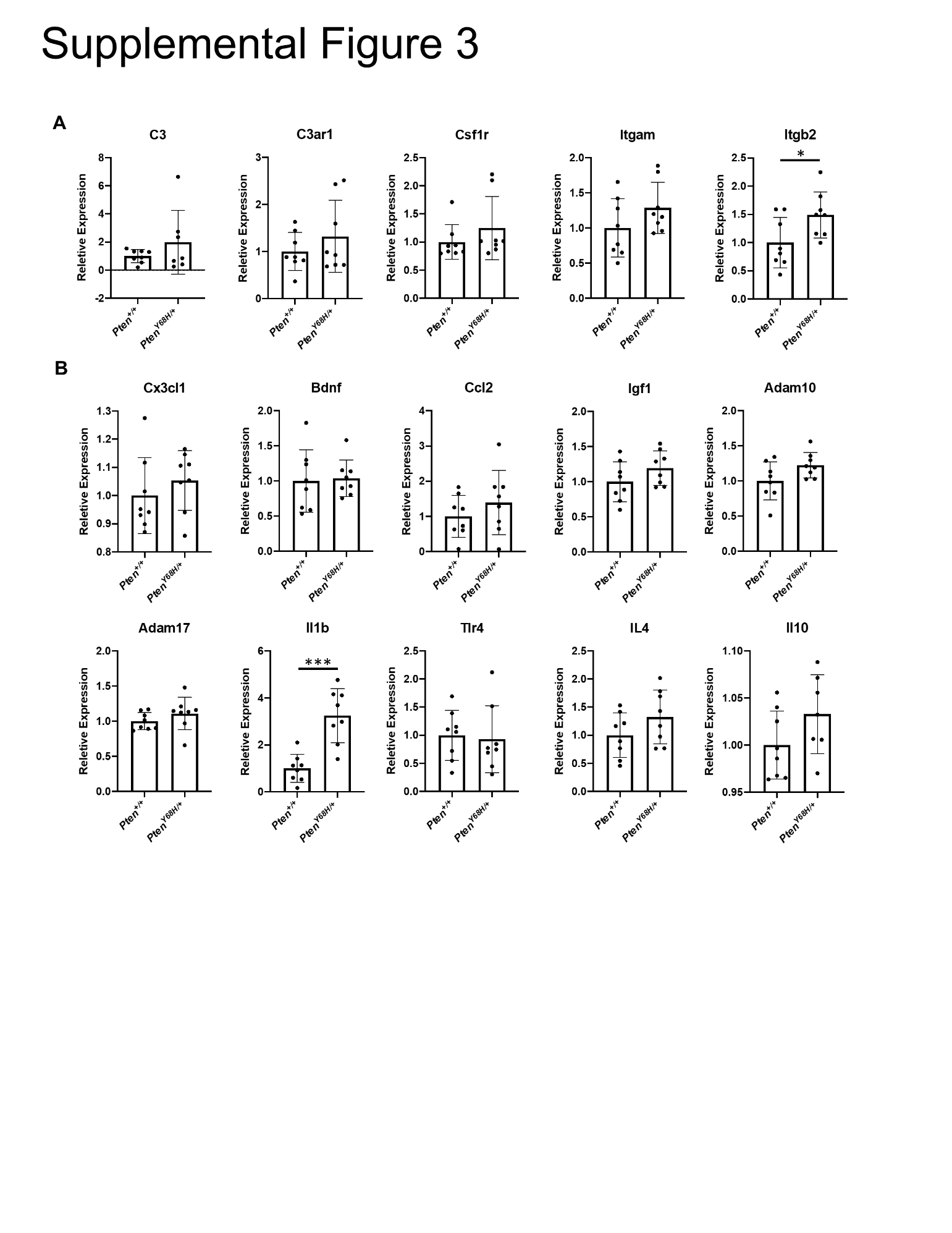


**Supplemental Figure S4.** Gene expression analysis with qRT-PCR on P40 *Pten^Y68H/+^* cortical RNA versus P40 wildtype cortical RNA. **a** qRT-PCR data on complement genes *C3, C3ar1, Csf1r, Itgam*, and *Itgb2*. **b** qRT-PCR data on fractalkine and immune signaling such as *Cx3cl1, Ccl2, Igf1, Adam10, Adam17, Tlr4, Il4,* and *Il10*.


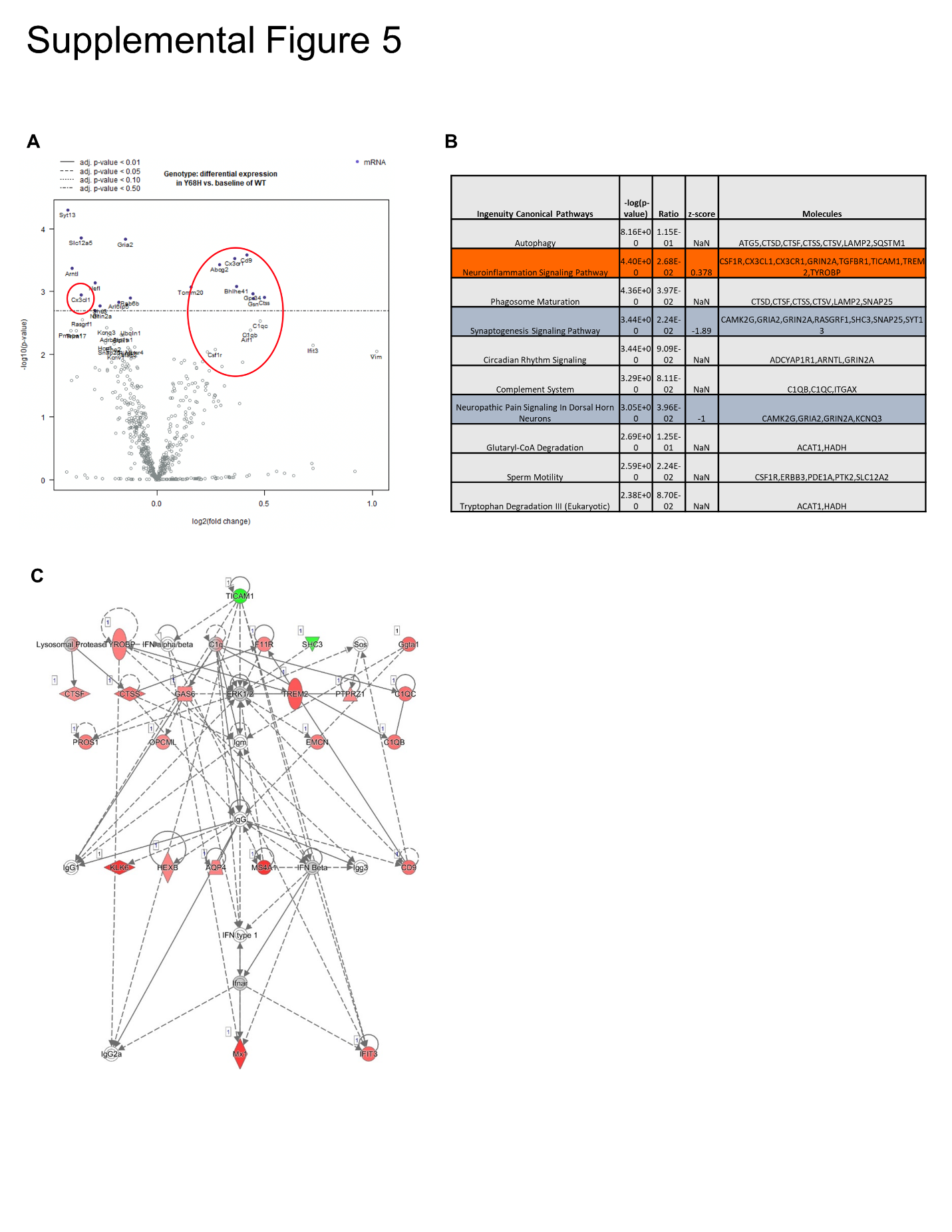


**Supplemental Figure S5.** NanoString glial gene expression panel analysis on P40 cortex from Y68H and wildtype mice. **a** Volcano plot showing differential gene expression. Microglia-related genes highlighted with red circles. **b** IPA canonical pathway analysis output for DE genes. **c** Top network identified by IPA. Green indicates downregulation. Red indicates upregulation. Solid line is a direct relationship. Dashed line is an indirect relationship.

**Table S1.** List of qRT-PCR primers

| **Gene Name** | **Forward Primer** | **Reverse Primer** |
| --- | --- | --- |
| *C1qa* | ATTCCCCTGGGTCTCCTTTA | ATGGGGCTCCAGGAAATC |
| *C3ar1* | TCTTGGGGTTGAAACAGAGG | AGTCCTGGAGCCTTTGGATT |
| *Cx3cr1* | CACTTGCCTCTGGTGGAGTC | AAGGAGGTGGACATGGTGAG |
| *C3* | GCAAGTGCTGACCAGTGAGA | CGTACTTGTGCCCCTCCTTA |
| *Itgam* | TTGGAAGGTCATGGGATGTT | TTCAGGGCCTTGTTCCTTTA |
| *Itgb2* | AGGCAAAGACATCTCCAGTCA | TCCCAGGAAGAACAGTCCAG |
| *Trem2* | GCTGAGGTCCTGCAGAAAGTA | CCTCGAAACTCGATGACTCC |
| *Dap12* | GACTGTGGTGTCCAGTGCAT | GGGCCTGTACGGGACTTAAT |
| *Csf1* | GACCATGGTGAATGGTAGGG | GCTTGCTAGGCTCCAATTTT |
| *Cx3cl1* | CGCGTTCTTCCATTTGTGTA | AGCTGATAGCGGATGAGCAA |
| *Adam10* | AAGATGGTGTTGCCGACAGT | TGAATCCACATTGTAAGATAATCC |
| *Adam17* | TCCTTATGGAGTGCTGCTGA | TCATCCTGACCACTTTGGTG |
| *Il1b* | TGTTCATCTCGGAGCCTGTA | GTGTCTTTCCCGTGGACCT |
| *Ccl2* | TCTCTCTTCCTCCACCACCA | TCATTGGGATCATCTTGCTG |
| *Igf1* | ACCACAGCTGGACCAGAGAC | CACTCATCCACAATGCCTGT |
| *Bdnf* | GGAGGTGGGGAATGGTACTT | TGAGTGTGGTTCTCCAATCG |
| *Il4* | TCAGTACTACGAGTAATCCATTTGC | AACTCTAGTGTTCTCATGGAGCTG |
| *Il10* | AAGGACCAGCTGGACAACAT | TCTCACCCAGGGAATTCAAA |
| *Tlr4* | GCTCCTGGCTAGGACTCTGA | CTTGGTTGAAGAAGGAATGTCA |

**Table S2.** *Pten^Y68H^* Transcriptome Total RNA Isolation Metrics.

| **Sample Name** | **Concentration After Total RNA Isolation (ng/uL)** | **A260/A280** | **A260/A230** | **A260** | **A280** | **Nucleic Acid Factor** | **Baseline Correction (nm)** | **Baseline Absorbance** | **28S/18S** | **RINe** | **Conc (ng/uL) for Sequencing** |
| --- | --- | --- | --- | --- | --- | --- | --- | --- | --- | --- | --- |
| Y68HWT1 | 1058.308 | 2.066 | 2.172 | 26.458 | 12.805 | 40 | 340 | 0.386 | 1.3 | 9.1 | 70.0 |
| Y68HWT2 | 2537.865 | 2.092 | 2.072 | 63.447 | 30.331 | 40 | 340 | 0.313 | 1.2 | 9.1 | 60.4 |
| Y68HWT3 | 2454.786 | 2.067 | 2.106 | 61.37 | 29.684 | 40 | 340 | -0.092 | 1.4 | 8.9 | 83.7 |
| Y68HWT4 | 2288.978 | 2.088 | 2.185 | 57.224 | 27.411 | 40 | 340 | 0.2 | 1.4 | 8.8 | 52.0 |
| Y68HWT5 | 2440.044 | 2.09 | 2.239 | 61.001 | 29.192 | 40 | 340 | 0.337 | 1.4 | 9 | 55.1 |
| Y68HWT6 | 2622.796 | 2.069 | 2.248 | 65.57 | 31.695 | 40 | 340 | -0.182 | 1.5 | 9.2 | 79.2 |
| Y68HWT7 | 2386.051 | 2.078 | 2.217 | 59.651 | 28.708 | 40 | 340 | 0.652 | 1.4 | 9 | 49.5 |
| Y68HWT8 | 2448.247 | 2.097 | 2.227 | 61.206 | 29.184 | 40 | 340 | 0.72 | 1.4 | 9.2 | 47.5 |
| Y68HHET9 | 2725.894 | 2.061 | 2.201 | 68.147 | 33.071 | 40 | 340 | -0.158 | 1.9 | 9.3 | 57.5 |
| Y68HHET10 | 2456.971 | 2.064 | 2.157 | 61.424 | 29.767 | 40 | 340 | -0.431 | 1.4 | 9 | 57.8 |
| Y68HHET11 | 2221.66 | 2.093 | 2.114 | 55.541 | 26.534 | 40 | 340 | 0.867 | 1.5 | 9.2 | 70.1 |
| Y68HHET12 | 2594.737 | 2.062 | 2.169 | 64.868 | 31.465 | 40 | 340 | -0.303 | 1.5 | 9.3 | 74.7 |
| Y68HHET13 | 2407.249 | 2.071 | 2.161 | 60.181 | 29.058 | 40 | 340 | -0.312 | 1.4 | 9.2 | 55.1 |
| Y68HHET14 | 2535.352 | 2.065 | 2.156 | 63.384 | 30.699 | 40 | 340 | -0.383 | 1.4 | 9.2 | 73.7 |
| Y68HHET15 | 2253.807 | 2.093 | 2.183 | 56.345 | 26.922 | 40 | 340 | 0.448 | 1.5 | 9.1 | 59.0 |
